# ApiAP2 Factors as Candidate Regulators of Stochastic Commitment to Merozoite Production in *Theileria annulata*

**DOI:** 10.1371/journal.pntd.0003933

**Published:** 2015-08-14

**Authors:** Marta Pieszko, William Weir, Ian Goodhead, Jane Kinnaird, Brian Shiels

**Affiliations:** 1 Institute of Biodiversity Animal Health and Comparative Medicine, College of Medical, Veterinary and Life Sciences, University of Glasgow, Bearsden Road, Glasgow, United Kingdom; 2 Institute of Integrative Biology, University of Liverpool, Crown Street, Liverpool, United Kingdom; Johns Hopkins Bloomberg School of Public Health, UNITED STATES

## Abstract

**Background:**

Differentiation of one life-cycle stage to the next is critical for survival and transmission of apicomplexan parasites. A number of studies have shown that stage differentiation is a stochastic process and is associated with a point that commits the cell to a change over in the pattern of gene expression. Studies on differentiation to merozoite production (merogony) in T. annulata postulated that commitment involves a concentration threshold of DNA binding proteins and an auto-regulatory loop.

**Principal Findings:**

In this study ApiAP2 DNA binding proteins that show changes in expression level during merogony of T. annulata have been identified. DNA motifs bound by orthologous domains in Plasmodium were found to be enriched in upstream regions of stage-regulated T. annulata genes and validated as targets for the T. annulata AP2 domains by electrophoretic mobility shift assay (EMSA). Two findings were of particular note: the gene in T. annulata encoding the orthologue of the ApiAP2 domain in the AP2-G factor that commits Plasmodium to gametocyte production, has an expression profile indicating involvement in transmission of T. annulata to the tick vector; genes encoding related domains that bind, or are predicted to bind, sequence motifs of the type 5'-(A)CACAC(A) are implicated in differential regulation of gene expression, with one gene (TA11145) likely to be preferentially up-regulated via auto-regulation as the cell progresses to merogony.

**Conclusions:**

We postulate that the Theileria factor possessing the AP2 domain orthologous to that of Plasmodium AP2-G may regulate gametocytogenesis in a similar manner to AP2-G. In addition, paralogous ApiAP2 factors that recognise 5'-(A)CACAC(A) type motifs could operate in a competitive manner to promote reversible progression towards the point that commits the cell to undergo merogony. Factors possessing AP2 domains that bind (or are predicted to bind) this motif are present in the vector-borne genera Theileria, Babesia and Plasmodium, and other Apicomplexa; leading to the proposal that the mechanisms that control stage differentiation will show a degree of conservation.

## Author Summary

The ability of vector-borne Apicomplexan parasites (*Babesia*, *Plasmodium* and *Theileria*) to change from one life-cycle stage to the next is critical for establishment of infection and transmission to new hosts. Stage differentiation steps of both *Plasmodium* and *Theileria* are known to involve stochastic transition through an intermediate form to a point that commits the cell to generate the next stage in the life-cycle. In this study we have identified genes encoding ApiAP2 DNA binding proteins in *Theileria annulata* that are differentially expressed during differentiation from the macroschizont stage, through merozoite production (merogony) to the piroplasm stage. The results provide evidence that the ApiAp2 factor in *Theileria* that possesses the orthologue of the *Plasmodium* AP2-G domain may also operate to regulate gametocytogenesis, and that progression to merogony is promoted by the ability of a merozoite DNA binding protein to preferentially up-regulate its own production. In addition, identification of multiple ApiAP2 DNA binding domains that bind related motifs within and across vector-borne Apicomplexan genera lead to the proposal that the mechanisms that promote the transition from asexual to sexual replication will show a degree of conservation.

## Introduction

The process of differentiation from one stage to the next is critical for survival, propagation and transmission of parasites within the phylum Apicomplexa. Differentiation steps can be conserved across genera. For example, generation of merozoites from an intracellular schizont, and the formation of gametocytes via merozoites that are committed for the sexual phase of the life-cycle, are events common to different members of the phylum. Moreover, differentiation steps across the Apicomplexa show a number of similarities indicating that the mechanisms involved are likely to have a degree of conservation. Apicomplexan stage differentiation events can occur in a stochastic manner (i.e. are asynchronous, with the probability of a differentiation step occurring influenced by culture/growth conditions and cell lineage) and are induced by multiple distinct stimuli [1,2]. In addition, work on *Plasmodium* and *Theileria* differentiation systems has provided evidence for an intermediate position, with progression towards or reversal from a point that commits the cell to generate the next life-cycle stage [3,4]. Drugs or conditions that alter the probability of a differentiation event occurring are likely to operate by altering the ability of a cell to reach a commitment threshold [5], and it can be hypothesised that the probability of switching from repeated rounds of asexual proliferation to the next phase of the life-cycle is governed by stage-determining commitment circuits that compete against each other, as identified in higher eukaryotic cell systems [6].

Candidates for Apicomplexan factors that control the switch in gene expression following a commitment to differentiate include members of the ApiAP2 gene family. ApiAP2 proteins were initially identified in the Apicomplexan genera *Cryptosporidium*, *Plasmodium* and *Theileria* [7], and have been subsequently identified in all Apicomplexan genomes analysed to date. All ApiAP2s possess an Apetala (AP2) domain of approximately 60 amino acids, originally identified as the DNA binding domain of transcription factors (TFs) that control developmental and stress-regulated gene expression in plants [8]. Work initially performed in *Plasmodium* has shown that ApiAP2s can bind to specific nucleotide motifs in the upstream regions of stage-regulated genes and are required to control their differential expression [9,10]. In addition, recent studies on *Toxoplasma gondii* have demonstrated the involvement of ApiAP2 factors in the regulation of the transition from the tachyzoite stage to the bradyzoite encysted stage [11,12] and they have also been shown to operate in commitment to gametocytogenesis in *Plasmodium* [13,14]. Identification of AP2 binding sites coupled with enrichment analysis of binding sites in stage-regulated genes has allowed prediction of networks that operate to control expression of ApiAP2 genes and their associated targets during the Intra-erythrocytic Developmental Cycle (IDC) of *P*. *falciparum* [9]. Moreover, the prediction that ApiAP2s regulate their own expression indicates they could operate in the stochastic model of stage differentiation previously proposed for *Theileria* [4].

*Theileria* is a tick-borne Apicomplexan parasite responsible for an economically important disease syndrome that threatens hundreds of millions of ruminants over large areas of the Old World. Currently, drugs are used as part of disease control strategies but emerging resistance against the most commonly used drug, buparvaquone, indicates that novel therapeutics will be required [15]. Based on the observation that the infection and treatment method of vaccination against *T*. *parva* operates by delaying differentiation to the intracellular macroschizont stage, targeting stage differentiation can be considered as a control strategy [5]. Previous work on *T*. *annulata* established an *in vitro* system of stage differentiation from the proliferating multi-nucleated macroschizont to production of the uni-nucleated merozoite (merogony). Analysis of this system established that differentiation is stochastic and that the probability of merogony occurring could be increased by inhibition of DNA synthesis, while inhibition of protein synthesis reduced the potential to reach commitment [16]. From these results it was postulated that during differentiation, an increase in the level of key DNA binding factors relative to their nucleic acid template occurs until a quantitative threshold, involving auto-regulation of gene expression, is reached that commits the cell to merozoite production [2]. Support for this model was provided by evidence for an increase in levels of factors in nuclear extracts of differentiating cultures that bind to a motif identified in the promoter region of the major merozoite antigen gene, *Tams1* [17]. In this study we have utilised microarray analysis to profile gene expression in *T*. *annulata* from the sporozoite stage through merogony to the piroplasm stage. Stage-regulated genes encoding AP2 DNA binding domains with orthologues in ApiAP2 factors of, primarily, related vector-borne genera (*Babesia* and *Plasmodium*) were then identified. Following this, cohorts of co-expressed genes were analysed to determine enrichment of nucleotide motifs bound by AP2 domains. The results identify ApiAP2 DNA binding domains (ApiAP2s) that are conserved across Apicomplexan genera and can be incorporated into a stochastic model of competitive factor binding that promotes reversible progression to the commitment point of stage differentiation.

## Methods

### Cell culture and mRNA isolation

Three cell lines were used in this study: the *T*. *annulata* infected D7 and D7B12 cloned cell lines provide a comparative *in vitro* system for merogony, as while D7 undergoes efficient differentiation to the merozoite when placed at 41°C, the D7B12 line (re-cloned from D7) is severely limited in its ability to differentiate under identical culture conditions [4]; BL20 is an uninfected bovine lymphosarcoma cell line [18]. Cell lines were cultured, induced to differentiate to the merozoite stage by increasing the temperature from 37°C to 41°C, harvested by centrifugation and total RNA isolated at Day 0, 4, 7 and 9 using Tri-reagent, as previously described [4,19]. RNA was also isolated using Tri-reagent from sporozoite-infected *Hyalomma* ticks and purified piroplasms, as described [20].

### Microarray and analysis

A whole-genome tiling microarray approach was used to investigate *T*. *annulata* gene expression during stage-differentiation. The most recent version of the *T*. *annulata* (Ankara C9) reference genome assembly and annotation [21], which was released in 2009 and is available at GeneDB (http://www.genedb.org/Homepage/Tannulata), was utilised to design a custom parasite microarray. The microarray consisted of abutting 45-mer oligonucleotide probes representing both DNA strands of each of the four nuclear chromosomes and the mitochondrial genome. The BLAST-like alignment tool (BLAT) [22] was used to match probe sequences to annotated spliced gene sequences. The sequence of each probe on the array was mapped to coding sequences utilising a flagging system similar to the web-based application, ProbeLynx [23]. A flag value of 1 represents a perfect, full-length alignment between a probe and gene, while a flag value of 5 represents poor alignment. For each individual probe, if a clear best match within the coding sequence was identified, that coding sequence (i.e. gene) was designated as the target of that probe and any poorer scoring BLAT-aligned sequences were designated as cross-hybridisation candidates. Only gene-specific probes were used in the present analysis, with flag thresholds based on previous experimental sensitivity and specificity studies of oligonucleotide arrays [24]. The array was designed for use on a 1,024 x 768 resolution chip and comprises 392,778 probes in total, 95% of which are targeted to the *T*. *annulata* genome. The remaining probes comprise bovine gene-targeted probes or control probes, including a set of over 15,000 oligonucleotides with random sequence and of mixed GC content. cDNA synthesis, labelling of cDNA and hybridisation to the microarray were performed by Roche NimbleGen. Parasite gene expression levels were determined using log_2_-transformed median intensity values and the data normalised using the Robust Multi-array Average [25]. The data discussed in this publication has been deposited in NCBI's Gene Expression Omnibus [26] and is accessible through GEO Series accession number GSE71307 (http://www.ncbi.nlm.nih.gov/geo/query/acc.cgi?acc=GSE71307). To determine whether a gene is expressed in a particular sample, the probe values for each gene were compared with background values from non-specific, random probes with equivalent GC content. Background hybridisation never exceeded a log_2_ intensity value of 10. Genes with a value of 10 or more were scored as expressed in a given parasite stage.

DNASTAR ArrayStar3 software was used to perform hierarchical clustering on log_2_-transformed gene expression levels. The results were visualised as a heat-map with the data clustered by sample (horizontally) and gene (vertically). Rank Product (RP) analysis is a non-parametric statistical test that may be used to identify differentially expressed genes between conditions using limited sets of replicates [27]. RP analysis was conducted on the following pair-wise comparisons: sporozoite to macroschizont, macroschizont (Day 0) to merozoite (Day 9), merozoite to piroplasm, and piroplasm to sporozoite. The obtained RP score was used to rank all the *T*. *annulata* genes in the dataset according to statistical confidence levels. Differentially expressed genes were assigned based on a false discovery rate (FDR) < 0.05 [28] and a fold change ≥ 2 (absolute). The same pipeline was used to generate a list of differentially expressed parasite genes between the D7 and D7B12 cell lines cultured at 37°C. Expression values for all identified *T*. *annulata* AP2 domain genes were then extracted from both datasets. Profiles of gene expression values (log_2_) across stages and during the differentiation time course were generated using DNASTAR ArrayStar3 software.

### Quantitative Reverse Transcription PCR

Two-step quantitative Reverse Transcription PCR (qRT-PCR) incorporating SYBR Green qRT-PCR methodology was utilised. cDNA synthesis was carried out in a total volume of 20μl using oligo(dT) primers and the method provided with the AffinityScript Multi-temperature cDNA Synthesis Kit (200436, Agilent). qRT-PCR reactions were performed using 500 ng cDNA template in a final volume of 25μl according to Brilliant SYBR Green QPCR Master Mix protocol (http://www.chem-agilent.com/) for real-time fluorescence detection of PCR product. To normalise qRT-PCR data, the genes encoding the heat shock 70 kDa protein (*TA11610*) and a heat shock 90 kDa protein (*TA10720*) were used based on constitutive expression from both microarray, semi-quantitative RT-PCR data and previous analysis by northern blotting [29]. Primers for both control and test genes were as follows: ***TA13515*** F, 5'-CGGGGAAGAGTGTAAAAATGAGTG and R, 5'-GGAGGTGATGGTCGTGATGG; ***TA11145*** F, 5'-CGTTGAGGGATCTTGTGAC and R, 5'-CTTCACACTCCTGTTCCCA; ***TA15705*** F, 5'-TGGAGATGGAGATAGCATGC and R, 5'-CTGGACCTCCAGATGCAC; ***TA11610*** F, 5'-ACGCAAATGGAATCCTCAAC and R, 5'-TATTCGTCGTGCTCTGCTAA; ***TA10720*** F, 5'-ACAATAGCAGAATCAGGAACAG and R, 5'-TATTGGGAAACGGATGAATTCTG; ***TA07100*** F, 5'-GCCACCCAGTAGACCTTCA and R, 5'-GTCGAGCATCAGCAAGTGT. Thermal cycling parameters used were: 1 cycle enzyme activation and initial denaturation, 10 min at 95°C; 40 cycles of PCR amplification (denaturation, 30 sec at 95°C; annealing, 60 sec at 60°C; elongation, 60 sec at 72°C); 1 cycle dissociation curve (60 s at 95°C, 30 s at 55°C and 30 s at 95°C). All qRT-PCR data was captured and analysed by MxPro v4.10 software with the Mx3005P Real-Time PCR System (Agilent Technologies). Melting curve analysis was carried out to verify product specificity and determine the presence of primer-dimers and other non-target products. Three technical replicates of each experimental time-point and no template controls were included in the PCR reactions for all sample points and primer sets. Expression values of target genes were normalised against Hsp70 (*TA11610*) and an Hsp90 gene (*TA10720*) and fold-change calculated relative to a calibrator point/condition, Day 0 –macroschizont stage, using a -2^-ΔΔCt^ equation [30]. Data was plotted as normalised mean values of log_2_ fold change ± the standard error of the mean (SEM). Statistical analysis was performed using a one-tailed Student’s t-test.

### PlasmoDB, BLAST, Motif enrichment analysis and MEME

Genes in *T*. *annulata* encoding orthologues of AP2 domains in related Apicomplexan species and genera were identified using BLASTP (www.ncbi.nlm.nih.gov/BLAST). ApiAP2 domain boundaries were defined as in Balaji *et al*. [7] and confirmed using the Pfam database (pfam.sanger.ac.uk). A cut-off of > 50% sequence identity was employed. Sequence alignments were generated using ClustalW (www.ebi.ac.uk/Tools/msa/clustalw2/). Alignment of all TaApiAP2s using T-coffee software (www.ebi.ac.uk/Tools/msa/tcoffee/) was generated to identify general conservation of the domain in *T*. *annulata*.

To establish whether upstream intergenic regions (IGR) of differentially expressed sets of genes were enriched for selected *Plasmodium falciparum* ApiAP2 domain target motifs, the motif pattern search (www.piroplasmadb.org/piro) function in PiroplasmaDB (version 1 and 2) was utilised. PiroplasmaDB was released in 2011 and is based on the pre-existing 2009 GeneDB assembly/annotation for *T*. *annulata*. A size restriction of 400 bp upstream of the predicted translation ATG start codon was employed, based on an average IGR length of 400 bp (with a large variance). This size was selected as IGRs are larger in a significant number of genes when flanked by the 5' boundary of predicted protein coding regions [31]. A search was also performed 100 bp upstream of the ATG, based on 5' un-translated region (UTR) size of 114 bp for the Tams1 gene (*TA17050*) of *T*. *annulata* [17]. Motif enrichment analysis was performed on the complete dataset of *T*. *annulata* predicted genes together with subsets of genes differentially expressed across stages and time points of the macroschizont (Day 0) to merozoite (Day 9) time course. The obtained data was exported to an Excel file and motif distribution data was tabulated. For each subset of genes, a motif enrichment P value was calculated by comparing the proportion of genes within the subset that possess the motif with the proportion of genes in a background list that possess the motif using a Fisher’s Exact Test. Pearson Correlation (positive or negative) of the expression pattern of genes which possess an ApiAP2 domain binding motif in their upstream region with the profile displayed by the gene encoding the ApiAP2 domain predicted to bind the motif was performed using Excel.

The Multiple Expectation Maximization for Elicitation of Motifs (MEME; version 4.6.1) software [32] was used to screen for putative motifs in IGRs of stage-regulated genes. Input sequences were prepared by extracting sequences upstream of predicted protein coding sequences (CDS) from the *T*. *annulata* GeneDB database. Searches were performed using a motif length of between 5 and 8 bp, 8 and 12 bp and 8 and 20 bp, and ZOOPS (Zero Or One Occurrence Per Sequence). The statistical significance of the motif was computed as an E-value based on an estimation of the expected number of motifs with the given log likelihood ratio and with the same width and site count that could be expected in a similarly sized set of random sequences.

To investigate the potential of ApiAP2 domains to bind to motifs in upstream of genes encoding the domain (auto-regulation), sequence alignments of upstream regions of selected *T*. *annulata* ApiAP2 genes to their *T*. *parva* orthologues was performed using ClustalW and visualised using Jalview. Alignments representing *TA13515*, *TA11145*, *TA12015* and *TA16485* were then searched for the core DNA binding motifs (5'-GTGTAC, 5'-CACACA/ACACAC, G-box/C-box or 5'-TCTACA) identified for the respective orthologous domain in *P*. *falciparum* [9] or *C*. *parvum* [33].

Based on previous phylogenetic analysis [7, 9], four TaApiAP2 domains (encoded by *TA11145*, *TA0710*, *TA19920* and *TA02615*) that could be predicted to bind to (A)CACAC(A) type motifs were selected and aligned to *T*. *parva*, *T*. *orientalis* and *P*. *falciparum* domain orthologues. Domain boundaries were defined using the Pfam database and a Maximum Likelihood tree constructed using RAxML [34]. Reciprocal BLAST analysis of each domain was also performed, as described above.

### Recombinant AP2 domain fusion proteins and parasite enriched nuclear extracts

Expression of selected AP2 domains as glutathione S-transferase (GST) fusion proteins was performed using the pGEX system. The regions selected for amplification included 10–20 nucleotides on either side of the sequence encoding each domain. Primers designed to create N-terminal GST-fusion constructs contained 5' and 3' extensions to create EcoRI and XhoI restriction sites respectively, for cloning. Primer sequences for each domain were: *TA13515* F, 5'-CAGGAATTCGTACAGGGTATGGTTGGATATTCT and R, 5'-GCACTCGAGGCTGAATACGCTCTACTGGAGTGC; *TA11145* F, 5'-CAGGAATTCCAAAGAACGAGCGCAAAGATTC and R, 5'-GTTCTCGAGTGTTAAATCTTATCATTATGTCTAAGTGC; *TA16485* F, 5'-CAGGAATTCAGAGCAAATTACTACCGAAGATTAG and R, 5'-GCACTCGAGCGGTCAGATTTGTTGGTTGGTTTCTG; *TA12015* F, 5'-CAGGAATTCTACCGAAGGAAGCCAATCTCATC and R, 5'-GCACTCGAGAGATGTGGTTCCTCTCGGT. PCR amplification was performed using the proof-reading Polymerase (Pfu) and *T*. *annulata* DNA (strain Ankara, clone C9) isolated from purified piroplasms. Amplicons were purified using a QiaQuick PCR Purification Kit (Qiagen, 28104), ligated into pGEX5x-2 vector DNA digested with EcoRI and Xhol, and competent XL-1 Blue cells (Stratagene, 200249) transformed using standard methodology. Recombinant clones were validated by DNA sequencing (Eurofins MWG Operon, Germany). Validated pGEX constructs were then re-transfected into BL21 Codon Plus (DE3)-RIL (Stratagene) competent cells and fusion proteins induced by IPTG (final concentration of 0.2 mM). Purification of fusion protein was performed using glutathione sepharose affinity beads (Sigma-Aldrich, GE17-0756-01) according to the manufacturer’s methodology. Protein concentrations were generated using the Better Bradford Assay Reagent, (Pierce Biotechnology, 23238). If required, GST-fusion proteins were concentrated using Amicon Ultra-15 Centrifugal Filter Units, with an Ultracel-3 membrane (Millipore, UFC900308). Eluted proteins were stored at -80°C in 25–50μl aliquots, at a concentration of 1 mg/ml.

Parasite-enriched Nuclear Extracts (PNE) were generated based on the method of Shiels *et al*. [17] but using the NE-PER Nuclear and Cytoplasmic Extraction Reagent kit, following the supplier’s instructions (Thermo Scientific). A differential centrifugation step (x 500 g to pellet host nuclei, followed by re-centrifugation of the supernatant at x 16,000 g) to enrich for parasite nuclei was incorporated after the initial cell lysis.

### Chemiluminescent Electrophoretic Mobility Shift Assay (EMSA)

To investigate protein-nucleic acid interaction, the Thermo Scientific LightShift Chemiluminescent Electrophoretic Mobility Shift Assay was employed. Single-stranded HPLC purified 5'-biotinylated oligonucleotides containing an ApiAP2 target or mutated motif (synthesised by Eurofins Genomics, Germany) were re-constituted in water to 100 pmol/μl. Labelled and complementary unlabelled oligonucleotides were annealed using a thermocycler in 20 mM Tris-HCl, pH 7.6; 50 mM NaCl, 10 mM NaCl at 50 μmol. Annealed oligonucleotides were diluted to 1 pmol/μl for non-labelled and 20 fmol/μl for biotinylated probes. For EMSA using fusion protein, in addition to the standard components used in the kit protocol, each reaction included 1 μl 50% glycerol, 1 μl 1% NP40, 2 μl fusion protein (0.7–1 mg/μl), 1 μl biotinylated oligo (20 fmol/μl). For reactions with PNE, additional components for optimisation were: 1 μl 50% glycerol, 1 μl 100 mM MgCl_2_, 1 μl 1% NP40, 1 μl EDTA, 5 μl of PNE, 2μl biotinylated oligo (40 fmol/μl). A 4% polyacrylamide gel was run at 100V, at 4°C and free and bound probes transferred to Biodyne Precut Nylon Membrane (Thermo Scientific, 77015) and then cross-linked at 120 mJ/cm^2^ using UV-light. Detection was performed using the Chemiluminescent Nucleic Acid Detection Module (Thermo Scientific, 89880). For competition experiments, cold double-stranded oligos were added to the reaction mix at 4–10 pmol and incubated on ice for 20 minutes before addition of the labeled probe. Oligonucleotides used as EMSA probes are listed (S1 Table).

## Results

### Identification of differentially regulated gene sets by microarray analysis

A microarray approach was utilised to profile gene expression of *T*. *annulata*. The array was screened with cDNA representing an *in vitro* stage-differentiation time-course from the macroschizont (Day 0) to cultures undergoing significant production of merozoites (Day 7 and Day 9), with an intermediate time-point (Day 4) included. The array was also hybridised with RNA representing sporozoites (the stage transmitted by ticks) that infect leukocytes and intra-erythrocytic piroplasms (the stage transmitted to ticks).

**Fig 1:**
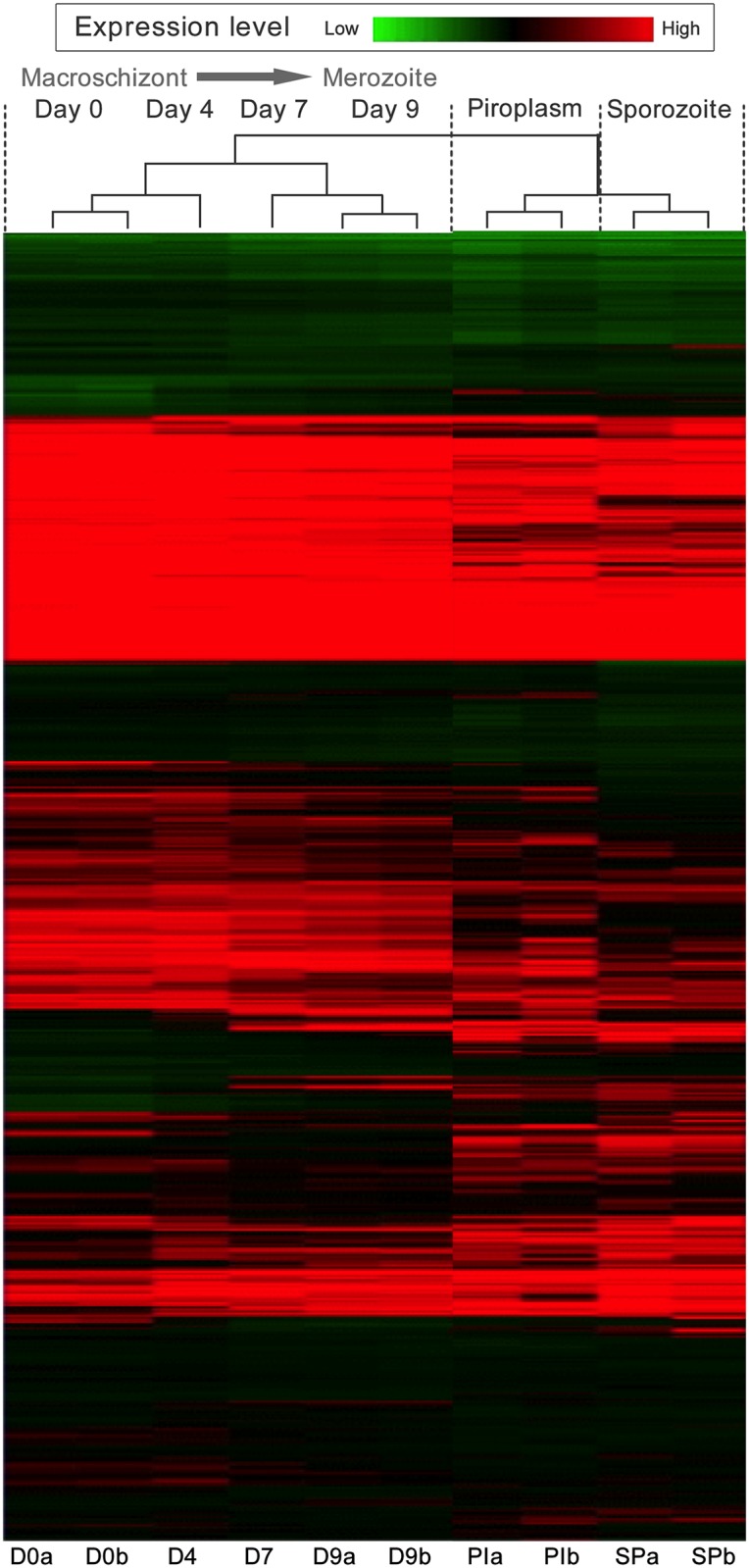
Life cycle stage and differentiation time course associated gene expression profiles in *T*. *annulata* identified by microarray analysis. Heat-map and hierarchical clustering of T. annulata gene expression profiles of 3,792 predicted protein-encoding genes generated by microarray analysis of RNA derived from a stage-differentiation time-course: Day 0 (macroschizont), Day 4, Day 7 and Day 9 (merozoite production (merogony) occurred at Day 7 and Day 9 time-points); piroplasms (PI) isolated from erythrocytes and tick-derived sporozoites (SP). Each horizontal line represents an individual gene, replicate samples are labelled a and b. Green bands represent genes expressed at low levels, while black and red bands represent intermediate and highly expressed genes respectively.

Hierarchical clustering was performed on log_2_-transformed gene expression data representing each *T*. *annulata* coding sequence (CDS). The data was clustered by sample and CDS, and is presented as a heat-map (Fig 1). This analysis showed that the Day 4 dataset clusters with Day 0, while the Day 7 data shows greatest similarity to the profile obtained for Day 9. However, with detailed inspection it can be seen that the day 4 time-point represents a transitional state, with genes that are down-regulated or up-regulated in the later time-points showing intermediate expression at Day 4: a few genes showed peak expression at this time-point. Significantly, clear differences in the expression level of genes were observed across different life-cycle stages and points of the *in vitro* differentiation time course e.g. Day 4 to Day 7. In addition, an appreciable number of genes, clustered at the top of the heat-map, show a pattern indicating constitutive expression. It can be concluded that a major change over in the control of gene expression occurs between Day 4 and Day 7 of differentiation to the merozoite *in vitro*.

Further analysis was performed using Rank Products (RP) to identify differentially expressed genes between different stages and time-points [27]. The numbers of genes identified for each pair-wise comparison are shown in Table 1. Datasets of the top 100 differentially expressed genes were generated, with further analysis focusing on the macroschizont (macro) to merozoite (mero) differentiation step. The up-regulated macro-mero list (S2 Table) is mostly comprised of genes encoding hypothetical proteins but also includes genes encoding rhoptry-associated proteins (*TA05870*, *TA05760* and *TA05705*), as predicted from previous studies [4,16]. Genes encoding a Map2 kinase (*TA21080*), cysteine protease (*TA04105*, *TA15660*), myosin (*TA20555*), a phosphate transporter (*TA13530*), a ubiquitin-conjugating enzyme E2 (*TA10690*), a cyclin-dependent serine/threonine kinase—related protein (*TA08470*) and an aspartyl (acid) protease (*TA17685*) were also identified as up-regulated during merogony. Three genes (*TA13515*, *TA16485* and *TA12015*) encoding proteins annotated as possessing AP2 DNA binding motifs were identified as significantly (FDR<0.05) up-regulated during differentiation to the merozoite.

**Table 1:** Number of genes displaying differential gene expression between stages identified by Rank Products (FDR<0.05).

Between-stage comparison	Up	Down
Sporozoite to macroschizont	66	133
Macroschizont to merozoite	152	115
Merozoite to piroplasm	24	20
Piroplasm to sporozoite	57	35

The list of down-regulated macro-mero genes (S3 Table) includes members of two gene families encoding proteins predicted to be secreted into the host cell compartment implicated in establishment of the macroschizont infected cell [35–37]. Thus, members of the SVSP family (e.g. *TA11410*, *TA09805*, *TA09790* and *TA09420*) and TashAT family genes (*TA2009*, *TA03125*, *TA03120*, *TA03145* and *TA03165*) were identified as highly down-regulated during differentiation to the merozoite. In addition, the gene encoding the macroschizont specific T cell antigen, Ta9 (*TA15705*) [38], was present in the list, as were members of the SfiI-subtelomeric fragment-related protein family and a gene (*TA10735*) encoding a putative GATA type transcription factor. Down-regulated expression (relative to the Day 0 (macroschizont) time-point) was validated for Ta9 by qRT-PCR with reduced expression most marked between the Day 4 and Day 9 time-point (S1 Fig).

### Expression profiles for *T*. *annulata* AP2 domain encoding genes

**Fig 2:**
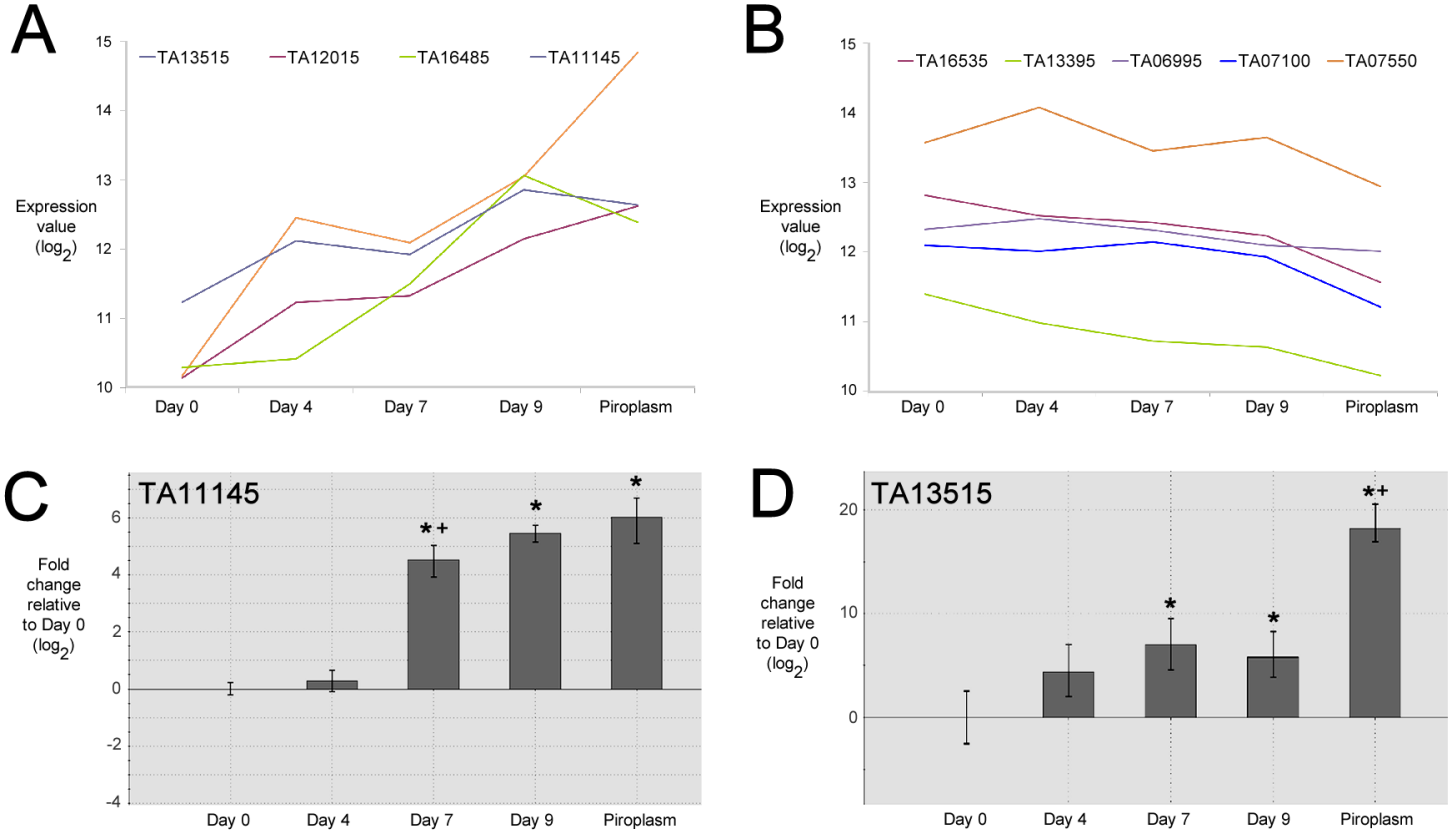
Temporal expression profiles of selected ApiAP2 genes in *T*. *annulata*. A. Microarray log_2_ expression values (Y-axis) of up-regulated ApiAP2 genes generated from RNA derived from an in vitro stage-differentiation time-course from Day 0 (macroschizont) to Day 9 (merozoite), and piroplasm stage (x-axis). B. Microarray log_2_ expression values (Y-axis) of down-regulated APiAP2 genes generated from RNA derived from an in vitro stage-differentiation time-course from Day 0 (macroschizont) to Day 9 (merozoite), and piroplasm stage (x-axis). C. QRT-PCR analysis of ApiAP2 gene, TA11145, expression, plotted as fold change in expression relative to the Day 0 (macroschizont) at Day 4, Day 7 and Day 9 (merozoite), and piroplasm stage. D. QRT-PCR analysis of ApiAP2 gene, TA13515, expression, plotted as fold change in expression relative to the Day 0 (macroschizont) at Day 4, Day 7 and Day 9 (merozoite), and piroplasm stage. Significant difference (P value ≤ 0.05) *, relative to Day 0 and +, relative to the preceding time-point/stage.

K means clustering was performed on log_2_-transformed gene expression data for all 22 ApiAP2 encoding genes in *T*. *annulata*. Two groups displayed expression profiles that could be associated with differentiation to the merozoite stage, i.e. showing generally progressive up-regulation and down-regulation respectively. The first of group of genes (Fig 2A) included the three AP2 domain genes identified by RP analysis as up-regulated during merogony. These genes were elevated, 7.36 fold (*TA13515*), 6.84 fold (*TA16485*) and 4.01 fold (*TA12015*) between Day 0 (macroschizont) and Day 9 (merozoite), with an additional ApiAP2 gene (*TA11145*) displaying a 3.08 fold increase between these time-points (FDR = 0.07). Notable differences in profile between these genes were, a higher relative level of expression in the macroschizont stage (Day 0) for *TA11145*, a delayed elevation in expression for *TA16485* (between Day 4 and Day 7) and a sustained, significant elevation in expression of *TA13515* through the Day 9 time-point (merozoite) to the piroplasm stage. Based on their temporal expression patterns we have denoted the factors encoded by these genes as TaAP2.me1 (*TA11145*), TaAP2.me2 (*TA12015*), TaAP2.me3 (*TA16485*); the fourth factor (encoded by *TA13515*) we have denoted as TaAP2.g based on elevated expression of the encoding gene in the piroplasm stage and high identity of the AP2 domain to the domain of the *Plasmodium* AP2-G factor (see below).

Genes in the second group possess a profile indicating reduced expression from the macroschizont stage (Day 0) through the merozoite stage (Day 9) to the piroplasm stage (Fig 2B). The change in expression level was not as marked but like the up-regulated group of ApiAP2 genes, differences between their profiles were manifest. For example, for *TA13395* a decrease in expression was observed between Day 0 and Day 4, whereas for *TA07550* expression increased from sporozoite though to the intermediate Day 4 time-point, followed by a reduction in levels at Day 7 and Day 9 (merozoite). *TA07100* did not display a reduction until between Day 9 (merozoite) and the piroplasm stage.

Of the four macroschizont to merozoite up-regulated AP2 genes, two, *TA11145* (TaAP2.me1) and *TA13515* (TaAP2.g), were selected for validation by qRT-PCR. The qRT-PCR results broadly supported the array data with significant up-regulation (p<0.05) relative to the Day 0 time-point at the Day 7 and Day 9 time-points and the piroplasm stage (Fig 2C and 2D). *TA11145* (TaAP2.me1) displayed elevation in expression level during merogony while for *TA13515* (TaAP2.g) the most significant increase in expression level was detected later, between the merozoite (Day 9) and piroplasm stage. In general, higher differences in expression levels between time-points were indicated by RT-PCR compared to microarray data, and a significant difference was detected in expression of *TA11145* between Day 4 and Day 7 that was not apparent with the array data. These differences are likely to arise from the increased quantitative sensitivity of qRT-PCR over the microarray platform, and inherent variability between differentiation time courses used to generate RNA for the two procedures [2].

### Conservation of *T*. *annulata* AP2 domains across related species and genera

**Fig 3:**
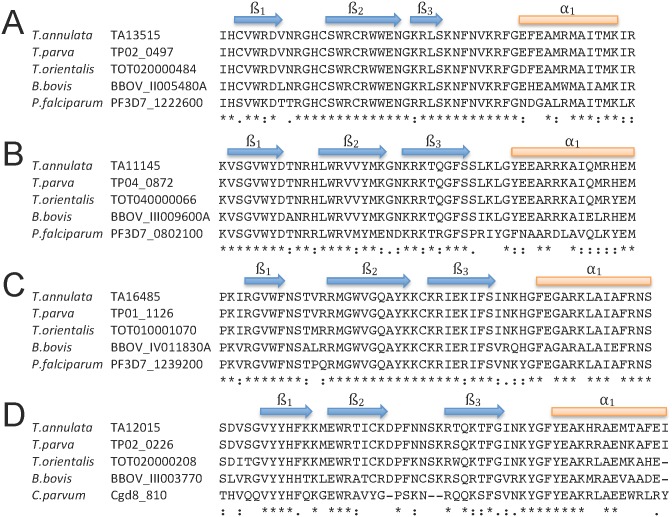
Alignment of *T*. *annulata* ApiAP2 domains, encoded by genes identified as up-regulated following merogony, with orthologous domains from related species and genera. A. Alignment of ApiAP2 domain encoded by TA13515 with orthologous domains identified by BLAST analysis from T. parva, T. orientalis, B. bovis, and P. falciparum or C. parvum: strong conservation of the domain across the related species is indicated (up to 100% identity) and genera (92% identity, 96% similarity with B. bovis; 80% identity, 94% similarity with P. falciparum). B. Alignment of ApiAP2 domain encoded by TA11145: strong conservation of the domain across the related species (100% identity) and genera (96% identity, 98% similarity with B. bovis; 72% identity, 82% similarity with P. falciparum) is apparent. C. Alignment of ApiAP2 domain encoded by TA16485: strong conservation of the domain across the related species (100% identity) and genera (96% identity, 100% similarity with B. bovis; 87% identity, 90% similarity with P. falciparum) is evident. D. Alignment of ApiAP2 domain encoded by TA12015: strong conservation of the domain across related species (91% identity with T. parva; 85% identity with T. orientalis), with strong to good conservation across genera (70% identity, 81% similarity with B. bovis; 52% identity, 68% similarity with C. parvum. Regions of predicted secondary structure are indicated above the alignment and were predicted by Phyre^2^ using three independent secondary structure prediction programs: Psi-Pred [58], SSPro [59] and JNet [60]. * identity,:. similarity.

Previous work has identified a considerable number of distinct consensus DNA motifs bound by different apicomplexan AP2 domains [9,39]. Moreover, it is known that ApiAP2 domain sequences can show conservation across Apicomplexan genera and that orthologous domains can bind closely related DNA motifs [9,12,39,40], although this is not always the case and domains that bind similar DNA motifs can show sequence diversity [39]. We, therefore, investigated conservation of the AP2 domain of the four *T*. *annulata* AP2 encoding genes that are up-regulated during merogony. Across *Theileria* species there is a high level of conservation in the primary structure of the AP2 domains within each of the four groups of orthologous domains with maintenance of the three anti-parallel beta strands and the alpha helix secondary structure (Fig 3). However, a degree of divergence between the four paralogous domains is evident (S2 Fig). In addition, for Ta.AP2.me1 (*TA11145*), TaAP2.me3 (*TA16485*) and TaAP2.g (*TA13515*), orthologous AP2 domains with strong identity were identified in *Babesia* and *Plasmodium* (Fig 3) species supporting previous studies [7], while for TaAP2.me2 (*TA12015*) AP2 domain orthologues were identified in *Babesia* and *Cryptosporidium* but not *Plasmodium*. Thus, it can be predicted that while orthologous groups of these AP2 domains may recognise similar DNA motifs, the four paralogous domains encoded by genes that are up-regulated during merogony in *T*. *annulata* are likely to recognise distinct motifs.

### DNA motifs predicted for *T*. *annulata* AP2 domains are enriched in upstream IGRs of stage-regulated genes

Based on identity across orthologues, data on the primary DNA motifs bound by *Plasmodium* AP2 domains [9] was utilised to investigate enrichment of these motifs in the *T*. *annulata* genome. *Plasmodium* orthologues (PF3D7_1222600 (previously, PFL1085w), PBANKA_143750) of TA13515 (TaAP2.g) encode the AP2-G factor critical for commitment to gametocytogenesis [13,14]. *Plasmodium* AP2-G binds the motif GxGTACxC, with GTAC identified as core nucleotides [9]: this motif was found to be significantly enriched (P < 0.0001) within a 400 bp region upstream of the ATG start codon on the positive strand of *T*. *annulata* genes up-regulated from merozoite to piroplasm (29% vs 4% of all other genes). No statistical enrichment of this motif was found in any other subset, implying this motif may be important for the up-regulation in expression of these genes from merozoite to piroplasm. The motif was also significantly enriched within 100–85 bp upstream of the ATG in the up-regulated merozoite to piroplasm gene set (25% vs 1.38%). This indicates the motif is either located within the 5' UTR or is just proximal to the transcription start site of genes with a UTR of less than 100 bp. A motif associated with genes enriched near telomeres and those encoding signal peptide proteins has been reported just proximal to the transcription start site in *T*. *parva* [31]. To validate that expression of genes enriched for the motif correlates with the expression profile of the gene encoding the AP2 domain predicted to bind the motif, the Pearson correlation coefficient value was computed. A perfect positive correlation (R = 1) was identified for *TA13515* (TaAP2.g) and the average profile of merozoite to piroplasm up-regulated genes possessing the motif (S3 Fig).

Enrichment for the core TCTAC(T)A motif bound by the *Plasmodium* orthologue (PF3D7_1239200 (PFL1900w)) of the AP2 domain of TaAP2.me3 (encoded by *TA16485*) indicated a possible association within 400 bp upstream of the ATG start codon of genes down-regulated from macroschizont to merozoite; 11.5% vs 6.7% of all other genes (P = 0.057). There was no significant enrichment indicated within the first 100 bp upstream of the translation start in the down-regulated gene set. A significant negative correlation (R = -0.92) was computed for the average profile of down-regulated macroschizont to merozoite genes enriched for the TCTAC motif and the expression profile of the *TA16485* (TaAP2.me3) gene (S3 Fig).

No direct orthologue of the AP2 domain encoded by *TA12015* (TaAP2.me2) can be identified in *Plasmodium*, but the domain orthologue in *C*. *parvum* (cgd8_810 Cpar) binds a G-box like motif [7,33]. A similar G-box like motif, A(G)NGGGGC(A) showed significant enrichment in the 400 bp upstream of the translation start site on the positive strand in IGRs of genes categorised as up-regulated from merozoite to piroplasm stage, with 45% vs 9% (P < 0.0001) of IGRs containing this motif. In addition, a significant depletion (1.7% vs 9.4%) was computed on the positive strand of upstream IGRs of genes down-regulated from macroschizont to merozoite (P < 0.005). The motif was not detected on the positive strand within 100 bp of the ATG start codon for either the down or up-regulated gene set.

The orthologue of the AP2 domain of TaAP2.me1 (*TA11145*) in *P*. *falciparum* is encoded by *PF3D7_0802100* (previously denoted, *MAL8P1*.*153*), which has been demonstrated to recognise a core motif rich in AC di-nucleotides [A/G]CACA[C/T][A/T] [9]. Although this motif type was commonly found within non-coding (intergenic) regions of the *T*. *annulata* genome, enrichment analysis found that there is a depletion of the motif in the 400 bp upstream region of IGRs of genes down-regulated from macroschizont to the merozoite stage, 13% vs 25% (P < 0.005). There was also evidence of enrichment 400 bp upstream of the ATG start codon in IGRs of genes up-regulated in merozoite, 31% vs 24% (P = 0.06) and piroplasm stages, 46% vs 24% (P < 0.05). No significant enrichment or depletion was obtained on analysis of the region 100 bp upstream of the translation initiation ATG codon. A positive Pearson Correlation (R = 0.93) was observed for expression of the TaAP2-me1 gene (*TA11145*) and the average profile of macro-mero most up-regulated gene set enriched for ACACAC in their upstream IGRs (S3 Fig). Analysis of 5' IGRs of genes upregulated from macroschizont to merozoite was also performed by MEME. The top motif identified was a 14 bp motif (AG)AATGTGTAA(AG)(GT)(TAG)(AT) (E-value = 1.3 x 10^−9^) with a conserved core motif of AATGTGTAA. This motif shows similarity with the reverse complement of the ACACAC motif, and identity with the motif previously identified by MEME in 5' IGRs of *T*. *parva* and *T*. *annulata* [31]. The motif has identity with a *P*. *falciparum* conserved TGTGT(G/A)(A/T) motif, and like its *Plasmodium* counterpart has a widespread distribution in non-coding regions of the genome. A role as a binding site for regulatory nuclear proteins other than transcription factors was proposed [31].

### Validation of motif binding by recombinant *T*. *annulata* AP2 DNA binding domains and factors in parasite-enriched nuclear extract

**Fig 4:**
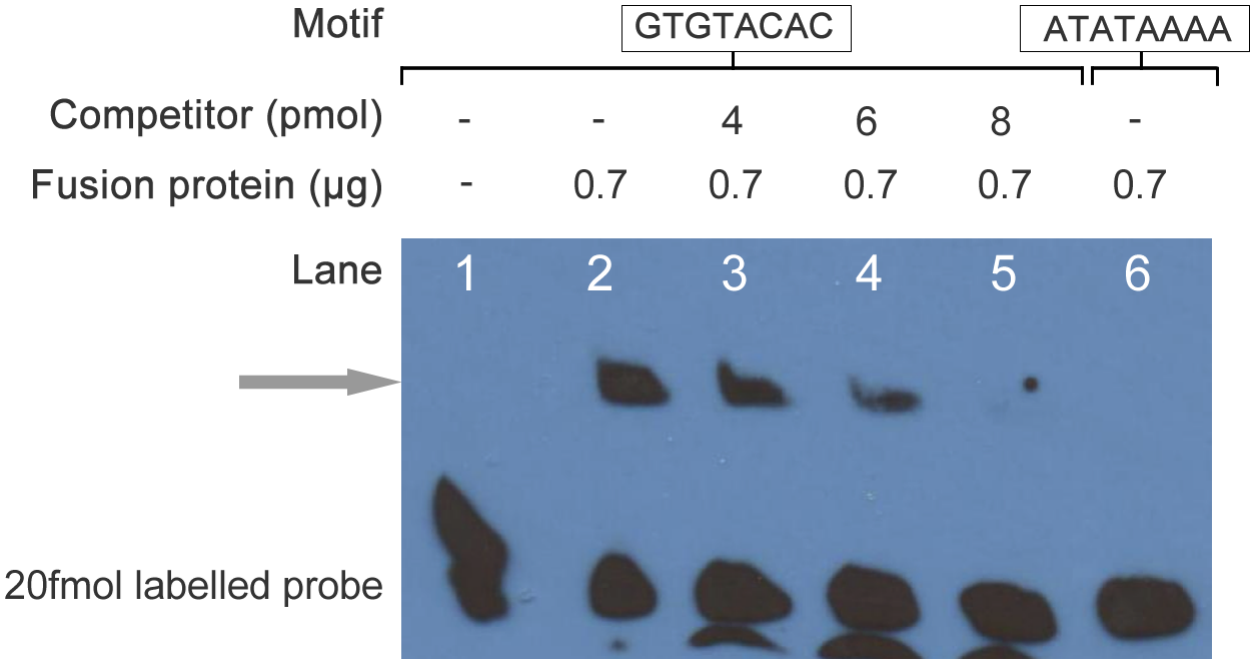
TA13515D AP2 domain (TaAP2.g) fusion protein domain binds to the motif identified for the orthologous AP2G domain of *Plasmodium*. EMSA performed with 0.7 μg of purified GST-TA13515D and 20 fmol of biotin-labelled double-stranded oligo probe containing the GTGTACAC motif recognised by the AP2 domain of Plasmodium AP2-G: lane 1, probe only; lane 2, probe + GST-TA13515D; lane 3, probe + GST-TA13515D + cold competitor (4 pmol); lane 4, probe + GST-TA13515D + competitor (6 pmol); lane 5, probe + GST-TA13515D + competitor (8 pmol); lane 6, mutated ATATAAAA probe (G/C in motif replaced with A) + GST-TA13515D; arrow indicates probe-specific shift.

To test whether *T*. *annulata* AP2 domains could bind the nucleotide motifs predicted for their *Plasmodium* orthologues, GST fusion proteins of the AP2 domain were generated for TaAP2.g (TA13515D), TaAP2.me1 (TA11145D), TaAP2.me2 (TA12015D) and TaAP2.me3 (TA16485D). The fusion proteins were then used in electrophoretic mobility shift assays against biotinylated double-stranded motif probes. As shown (Fig 4), recombinant AP2 DNA-binding domain of TaAP2.g (TA13515D) strongly bound to the probe representing the consensus core motif, GTGTACAC (GxGTACxC) bound by orthologous AP2-G domains of *Plasmodium* [13,14]. The shift complex was competed with unlabelled probe, and no shifts were obtained using a mutated core binding site (G/C replaced with A in motif) probe (Fig 4, lane 6).

Similar results were obtained for the recombinant AP2 domain of TaAP2.me1 (TA16485D), as a shift was obtained using a probe containing the core motif TCTACA identified for the orthologous *P*. *falciparum* domain [9]. The EMSA generated a clear band shift (S4 Fig) with binding specificity indicated by a reduction in the detected shift on addition of cold probe. EMSA with the AP2 domain encoded by *TA12015*, predicted to bind a G box like motif, did not generate a detectable shift (S5 Fig).

**Fig 5:**
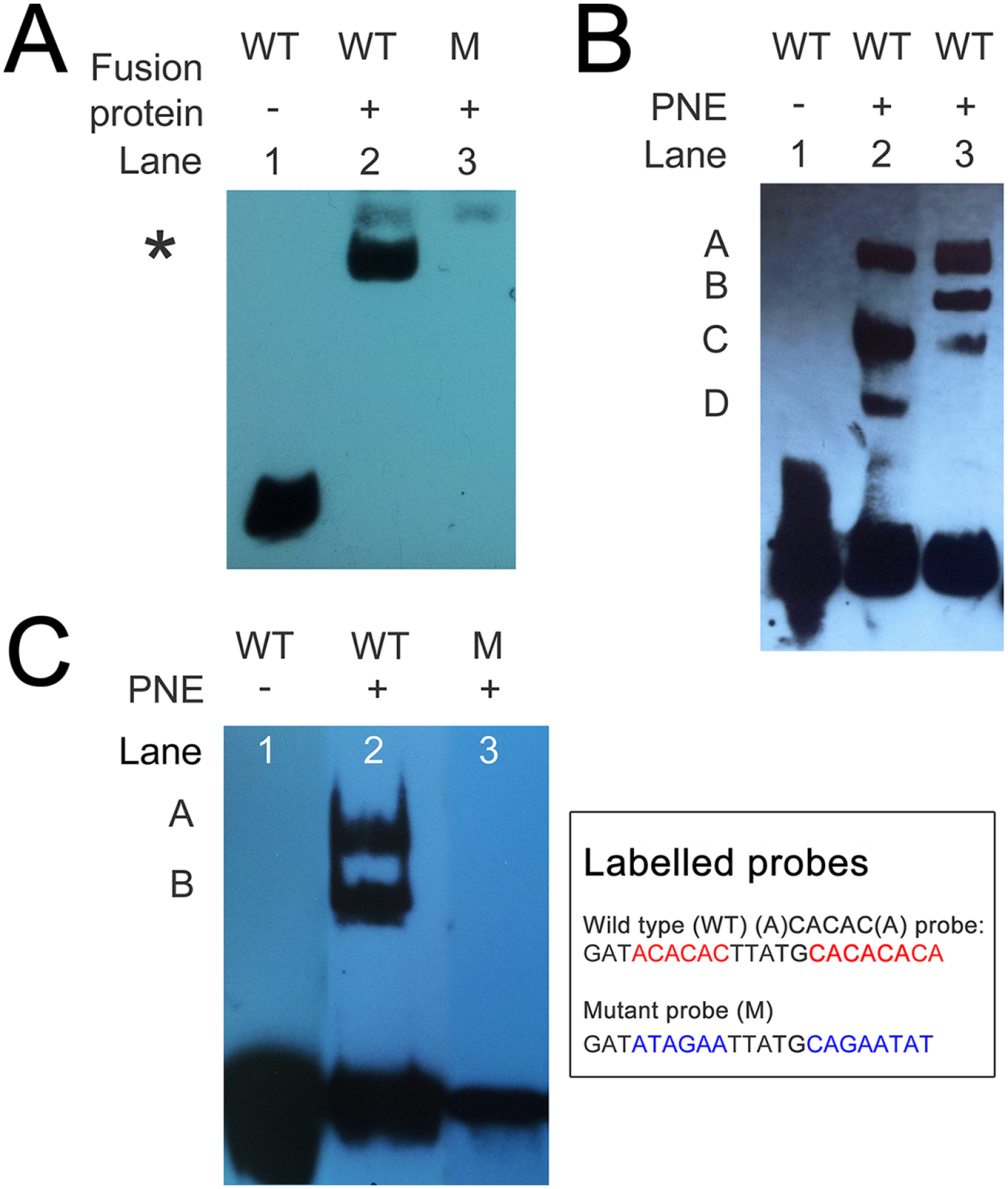
TA11145D AP2 domain (TaAP2.me1) fusion protein and PNE factor(s) bind to an (A)CACAC(A) type motif upstream of the TA11145 gene. A. EMSA performed with 0.7μg of purified GST-TA11145D and 20 fmol of biotin-labelled double stranded oligo probe containing a double (A)CACAC(A) motif: lane 1, probe alone; lane 2, probe + GST-TA11145D; lane 3, mutant probe with both motifs mutated * denotes position of specific shift. B. EMSA performed with PNE and the double (A)CACAC(A) motif probe: lane 1, probe alone; lane 2, probe + PNE Day 0; lane 3, probe + PNE Day 9. Letters denote shifts detected, shift B was only present in Day 9 PNE. C. EMSA performed with PNE, and above probe: lane 1, probe only; lane 2, probe + PNE Day 9; lane 3, mutated probe + PNE Day 9, the infection associated shift detected in Day 9 PNE was not obtained with the mutant probe.

The *P*. *falciparum* orthologue of the AP2 domain of TaAP2.me1 (*TA11145*) has been shown to bind the motif ACACAC [9]. To test that TaAP2.me1 could also bind this motif, EMSA was performed using the AP2 domain fusion protein and a probe containing a double ACACAC type motif located in the intergenic region upstream of the encoding gene (*TA11145*). The TaAP2.me1 AP2 domain fusion protein (TA11145D) generated a strong shift with this probe (Fig 5A). To confirm that binding required the ACACAC motifs, these motifs were mutated. A shift was not observed with the mutated probe. Thus, the TaAP2.me1 AP2 DBD has the capacity to bind specifically to a double motif in the upstream region of its own encoding gene (*TA11145*).

To determine if native nuclear factors could bind to the probe representing the double ACACAC type motif,, EMSA was performed using extracts from parasite-enriched nuclear fractions derived from macroschizont-infected cells (Day 0) and infected cells undergoing differentiation to the merozoite (Day 9). Fig 5B shows that EMSA performed with extracts derived from parasite-enriched nuclear extracts generated a total of 4 shift complexes A-D. Shifts A, C and D were also detected with nuclear extracts derived from uninfected host BL20 cells (S6 Fig) and were concluded to be derived from host contamination in PNE. Shift B, however, was only obtained using extracts derived from cultures undergoing merozoite production and was not detected in host-derived nuclear extracts. To confirm that the up-regulated B shift required the (A)CACAC(A) motifs, EMSA was performed using the mutant probe and Day 9 PNE (Fig 5C): the up-regulated shift B was not obtained with this probe. The results indicate that a nuclear factor(s) associated with cultures undergoing differentiation to the merozoite can specifically bind to (A)CACAC(A) motifs upstream of the *TA11145* gene that is up-regulated during merogony.

### AP2 domain genes up-regulated during differentiation to the merozoite are predicted to auto-regulate

Demonstration that the ACACAC motif in the IGR upstream of the *TA11145* gene is recognised by the AP2 binding domain encoded by the gene indicates that its expression may be auto-regulated. This is of interest as the stochastic model of merogony in *T*. *annulata* predicted that the commitment to differentiate is reached via the capacity of regulators of differential gene expression to auto-regulate. It was investigated, therefore, whether there is a greater occurrence of the motif in the IGR upstream of the domain encoding *TA11145* gene relative to the other 21 AP2 encoding genes. Seven ACACAC type motifs were found in the IGR upstream of *TA11145* (including the double motif separated by five nucleotides) and six were conserved upstream of the *T*. *parva* orthologue (S7 Fig). In contrast, a maximum of three motifs were detected in the IGR upstream of two other AP2 domain genes (*TA05055* and *TA08375*) and an average of 0.95 motifs per AP2 gene IGR was obtained. Both ApiAP2 genes with three motifs were classed in the same expression profile as *TA11145* (up from macroschizont (Day 0) to merozoite (Day 9)).

Auto-regulation has also been predicted for genes encoding the *Plasmodium* AP2G factor (*PF3D7_1222600*, *PBANKA_143750*) [13,14]. Screening for the core motif (GTAC) bound by the TaAP2.g domain detected it’s presence at three positions in the upstream intergenic region of the encoding gene (*TA13515*), including a double motif separated by 6 bp (core A to core G). These three motifs were conserved in the upstream region of the *T*. *parva* gene encoding the orthologous domain (*TP02_0497*) [41].

### Multiple AP2 domains are predicted to bind (A)CACAC(A) type motifs in *T*. *annulata* and *Plasmodium*

**Fig 6:**
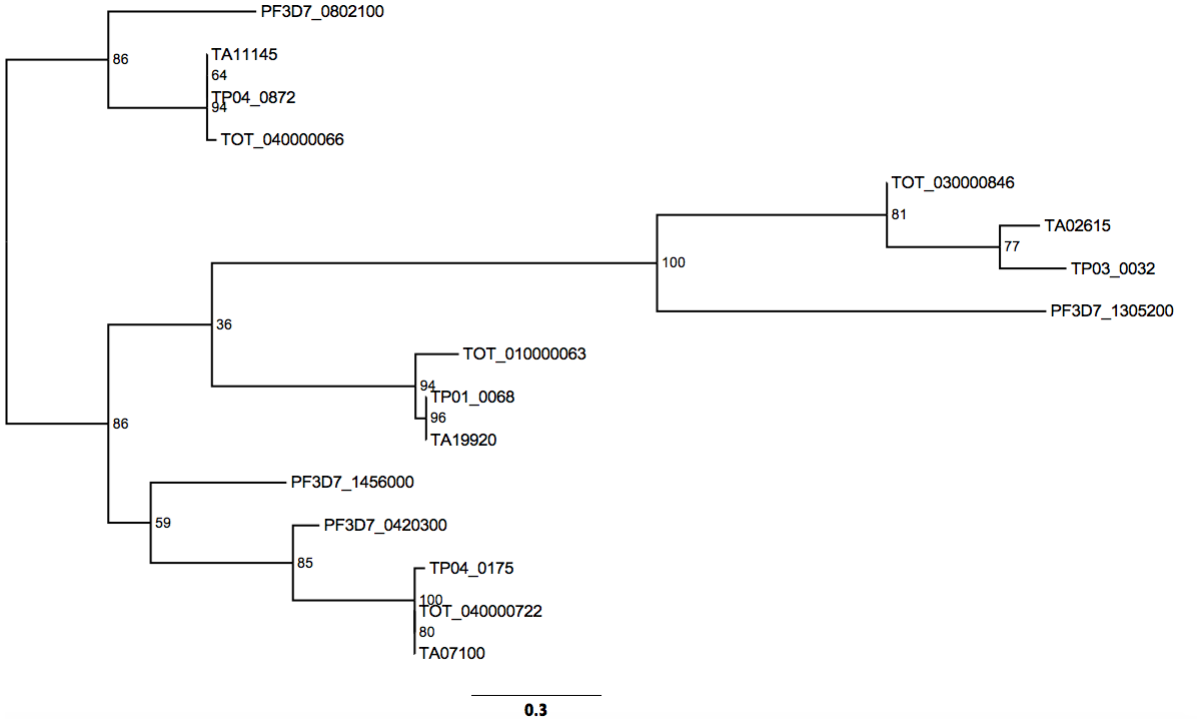
Phylogenetic tree of AP2 domains from *P*. *falciparum* and *Theileria* orthologues predicted to bind to (A)CACAC(A) type motifs. A maximum likelihood phylogenetic tree was constructed using the amino acid sequence of AP2 domains of related genes in T. annulata, T. parva, T. orientalis and P. falciparum. Three clusters of orthologous domains with a representative from each species can be observed for TA11145, TA02615 and TA07100. No clear Plasmodium orthologue was detected for the TA19920 domain. Percentage bootstrap values are shown at each node on the tree.

In *P*. *falciparum*, multiple AP2 domains have been shown to bind to motifs rich in CA di-nucleotides, two variants being ACACAC and CACACA [9]. We term these (A)CACAC(A) type motifs, where an A is present either at the 5', 3' or both ends of the motif. *Theileria* orthologues of *Plasmodium* AP2 domains that bind (A)CACAC(A) can be identified in the phylogenetic analysis performed by Balaji *et al*., [7]. Thus domains encoded by *TA11145*, *TA07100* and *TA02615* were found to be orthologues of the domains encoded by *PF3D7_0802100* (*MAL8P1*.*153*), *PF3D7_0420300* (*PFD0985w*.D1) and *PF3D7_1305200* (*PFL13_0026*), while the domain encoded by *TA19920* was placed in a position in the tree intermediate between TA07100 and TA11145 but without a clear domain orthologue indicated in *Plasmodium*. To analyse this group of domains in more detail, a maximum likelihood tree was constructed with the four *P*. *falciparum* domains and the putative orthologous domains from *T*. *annulata*, *T*. *parva* and *T*. *orientalis* (Fig 6). The tree generated essentially supports the phylogeny of Balaji *et al*. [7] with three clear orthologous groups, containing TA07100, TA11145 or TA02615 domains, indicated. The domains encoded by *TA19920* and a fourth *Plasmodium* (A)CACAC(A) binding domain, encoded by *PF3D7_1456000* (*PF14_0533*), did not fit into an orthologous group. This was supported by reciprocal BLAST analysis with no clear orthologue identified for the TA19920 AP2 domain in *Plasmodium* or the PF3D7_1456000 domain in *Theileria*.

An alignment of AP2 domains in the orthologous groups represented by TA11145 and TA07100 domains, respectively (Figs 3B and S8) indicates that these domains are highly likely to bind related (A)CACAC(A) motifs in *Babesia*, *Theileria* and *Plasmodium*. Thus, there are, at least, two phylogenetically related AP2 domains conserved in vector-borne Apicomplexa that bind (A)CACAC(A) type motifs, with orthologous members of a third ApiAP2 domain (represented by TA02615) possibly binding to this, or a closely related, motif.

### *TA11145* gene expression is significantly elevated in the D7 infected cell line compared to a line severely attenuated for differentiation to the merozoite

**Fig 7:**
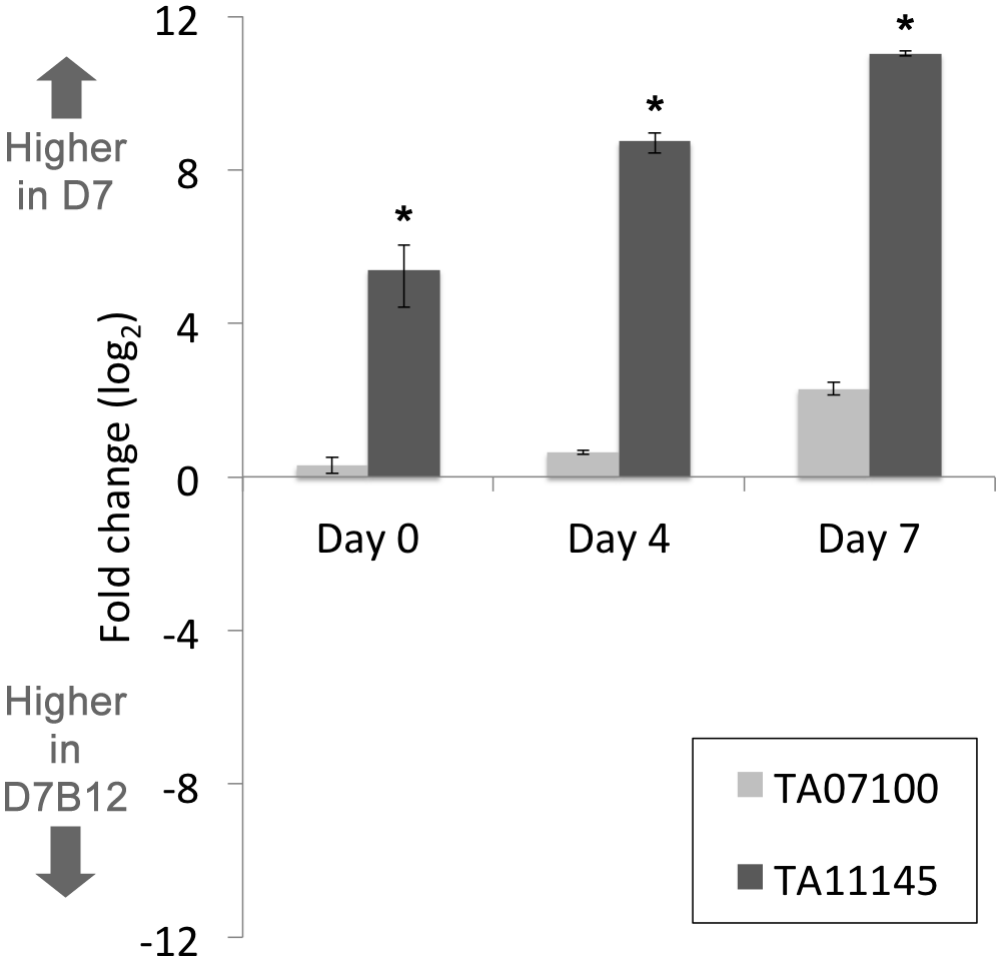
Expression of *TA11145* (TaAP2.me1) is significantly higher in the D7 cell line compared to a cell line that has lost the ability to differentiate to the merozoite. A. QRT-PCR data plotted as fold change in elevated expression (log_2_) for ApiAP2 domain encoding genes TA11145 and TA07100 in the differentiation competent D7 cell line versus the attenuated D7B12 cell line. Fold change in expression between cell lines was computed at Day 0 (macroschizont) and Day 4 and Day 7 points of a time-course of differentiation to the merozoite; * denotes significant (P value ≤ 0.05) fold change elevated expression in D7 vs D7B12.

Cell line D7B12 is severely attenuated in its ability to undergo differentiation to the merozoite stage [4]. However, merogony is not totally abrogated and it can be postulated that the attenuated phenotype may be linked to a quantitative alteration in expression of key regulatory molecules. To test whether this might be associated with AP2 domain encoding genes, microarray data was generated for the D7B12 line (Day 0) and compared to data for the differentiation competent D7 line (Day 0). Three AP2 domain genes were predicted to show significantly higher expression in D7 relative to D7B12 (S4 Table). One of these genes (*TA11145*) encodes the domain shown to bind (A)CACAC(A) and is up-regulated during merogony; while the gene (*TA07100*) encoding the other AP2 domain in *T*. *annulata* that is strongly predicted to bind (A)CACAC(A) did not show a significant difference. To validate the difference in expression qRT-PCR was performed for the *TA11145*, and *TA01700* genes using RNA derived from D7 and D7B12 cells cultured at 37°C (Day 0) and during progression to merogony at 41°C (Day 4 and Day 7). The results indicate that RNA levels of *TA11145* were significantly higher (5.4 fold, log_2_) in D7 vs D7B12 cells at the Day 0 time-point and that this difference is exacerbated following culture at 41°C: 8.7 fold (log_2_) at Day 4 and 11 fold (log_2_) at Day 7 (Fig 7). In contrast, qRT-PCR performed for *TA07100* showed a relative difference of 0.2 fold, 0.6 fold and 2.2 fold (log_2_) higher in D7 cells at Day 0, Day 4 and Day 7, respectively. This validates that the TaAP2.me1 gene (*TA11145*) is expressed at a higher level in a cell line competent for differentiation and that up-regulation is independent of a heat shock response. Thus, the expression level of *TA11145* relative to *TA07100* is clearly altered in favour of *TA11145* in the D7 cell line and this bias is increased during progression towards merogony (Day 4 and Day 7).

## Discussion

Within the life cycles of Apicomplexan parasites, transition from stages that undergo multiple rounds of asexual replication to stages that promote life-cycle progression and parasite transmission are regulated by critical cellular differentiation events. Evidence generated across apicomplexan genera indicates that these transition points operate on a stochastic basis and that stage-differentiation steps can be programmed to occur in a time-dependent manner [1,2,13,14,42]. Together, these findings indicate that a basic mechanism may have been conserved.

From previous investigation of merogony in *T*. *annulata in vitro* it was proposed that the stochastic differentiation mechanism involves the build up of DNA binding protein(s) relative to their DNA template to generate a commitment point involving an auto-regulatory loop [2]. The aim of the present study was to identify potential DNA binding factors and nucleotide motifs that could play a role in this differentiation model.

To search for motifs and DNA binding factors associated with stage differentiation, expression data representing the sporozoite stage, a macroschizont (Day 0) to merozoite stage (Day 9) differentiation time-course, and piroplasm stage was generated. Comparative analysis produced two sizeable lists of genes up- or down-regulated during differentiation to the merozoite (152 and 115 at FDR < 0.05, respectively). Both lists contained genes predicted from previous studies. For example, genes encoding rhoptry proteins, Tams1 [4] and cysteine proteinases [43] were defined as up-regulated whereas members of the TashAT and SVSP encoding gene families, implicated in establishment of the proliferating macroschizont-infected cell [36,37], were identified as down-regulated during differentiation to the merozoite.

Several general observations could be made from the temporal expression patterns. Firstly, genes expressed at a high level in one stage were often indicated as expressed at a low level in the preceding or subsequent stage. This supports previous studies reporting that merozoite genes are expressed at the macroschizont stage [4], and that a low level of non-stringently regulated mRNA expression operates in *T*. *parva* [44]. Thus, repression of gene expression in a stage-specific manner at the mRNA level is unlikely to be absolute in *Theileria*. The data also indicated differences in the pattern of expression for distinct genes across the differentiation (merogony) time-course, implying regulation of gene expression via multiple factors that operate in a temporal order. Similar results have been reported for related Apicomplexa [9,33,45] and cascades of transcriptional regulators proposed for these systems are likely to operate for *T*. *annulata*.

Recent studies on DNA binding proteins have provided strong evidence of their involvement in the regulation of Apicomplexan stage-differentiation events. The *T*. *annulata* microarray data was therefore screened for predicted DNA binding proteins that showed differences in expression level between macroschizont and merozoite stage. Genes encoding four putative AP2 domain DNA binding proteins were found to show elevation of mRNA levels between macroschizont and merozoite.

Comparison of the four up-regulated *T*. *annulata* AP2 domain amino acid sequences with the orthologous domains in *Plasmodium* or *Cryptosporidium* (TA12015) showed that there was greater identity for orthologues across genera than between these four paralogous domains within *T*. *annulata*. This allowed prediction that each of the four domains bound different target motifs. In addition, consensus motifs bound by the AP2 domains in *Plasmodium* or *Cryptosporidium* were predicted for the orthologous domain in *T*. *annulata*. EMSA performed with fusion protein domains encoded by *TA11145*, *TA13515* and *TA16485* demonstrated that this prediction was valid. Based on previous studies [9,39,40] this does not mean that the AP2 factors from different genera operate to control gene expression in the same life-cycle stage or regulate the same genes, because orthologous domains across genera have been shown to target different gene sets. Our results for domains encoded by *TA13515* and *TA11145*, however, do show parallels with data obtained for their orthologues in *Plasmodium*.

The direct orthologue of the AP2 domain encoded by *TA13515* in *Plasmodium* is the domain of the AP2-G factor that is essential for commitment to gametocyte production [13,14]. The AP2 domain of AP2-G shows a high degree of conservation with the domain encoded by *TA13515* (92% identity) and binds the motif GxGTACxC. As expected, the *Theileria* AP2.g domain specifically bound the GxGTACxC motif. This motif is enriched in the upstream region of genes up-regulated from merozoite to piroplasm stage in *T*. *annulata*, with no enrichment in any other subset of stage-regulated genes. Furthermore, in a similar manner to *Plasmodium* AP2-G, a GTAC core motif is present in three copies (one double motif) in the upstream region of the TaAP2.g gene (*TA13515*) and *T*. *parva* orthologue (*TP02_0497*), indicating putative auto-regulation of gene expression. A role in regulating gene expression as the parasite differentiates into the piroplasm stage is highly likely. The piroplasm stage has been postulated to be equivalent to gametocytes and it is known that a sexual phase occurs within the tick [46]. Thus, as recently suggested, orthologues of AP2-G could contribute to sexual stage switching across vector borne Apicomplexa and provide a target for transmission blocking strategies [47]. AP2-G expression and gametocytogenesis has been associated with a stress response in *Plasmodium* [48]. The expression profile obtained for *T*. *annulata*, however, indicates that up-regulation is primarily liked to developmental events. This conclusion is supported by demonstration of significantly elevated expression in the D7 cell line vs D7B12 cell line when both lines were cultured for 7 days at 41°C (S9 Fig).

The AP2 domain encoded by *TA11145* (TaAP2.me1) is the orthologue of the PF3D7_0802100 (MAL8P1.153) domain in *P*. *falciparum* and TGME49_071030 in *T*. *gondii* [39], and domain orthologues with high identity are present in *Babesia* (Fig 3). Our findings allow postulation that *TA11145* is a key regulator of stochastic commitment to merozoite production in *T*. *annulata*. The gene is expressed at the RNA level at the preceding stage of the life-cycle and shows significant elevation during the differentiation time-course. Moreover, expression is significantly reduced in a cell line that has lost the ability to differentiate to the merozoite. Motifs recognised by the AP2 domain encoded by *TA11145* are the (A)CACAC(A) type motifs detected by its orthologous domain in *Plasmodium* [9]. This motif is common in the non-coding region of the genome, but showed evidence of being enriched in the upstream IGRs of genes up-regulated during merogony, while depleted in upstream regions of down-regulated genes. The motif type is also over-represented in non-coding regions of the *Plasmodium* and *Toxoplasma* genomes [31,49,50], but was observed to be associated with a large group of genes expressed during the middle to later stages of the Intra-erythrocytic Developmental Cycle (IDC) of *P*. *falciparum* [50]. In addition, this motif type is recognised by two AP2 factors critical for regulation of tachyzoite to bradyzoite conversion in *T*. *gondii* [11,12], one of which possess a AP2 domain that is the orthologue of the domain encoded by *TA07100* in *T*. *annulata* and *PF3D7_0420300* (*PFD0985w*.D1) in *P*. *falciparum* [39]. The motif may have a general role in genome organisation associated with differential gene expression, possibly acting as a site for accessory factors that modulate chromatin structure.

In *Plasmodium*, four AP2 domains have been shown to recognise (A)CACAC(A) type motifs, three of which are closely related to each other [9]. Expression of the genes encoding these three domains occurs at different points of the IDC, and two (*PF3D7_0802100* and *PF3D7145600*) are predicted to auto-regulate [9]. Based on phylogenetic analysis, a similar situation exits for *Theileria*, with at least two domains (encoded by *TA11145* and *TA07100*) displaying a level of similarity to their *Plasmodium* orthologues that indicates binding to the same or similar motif. Auto-regulation is predicted for *TA07100* and *TA11145* (and potentially *TA02615*). However, since there is only one (A)CACAC(A) motif upstream of *TA07100* relative to seven in *TA11145* there is a much stronger prediction of auto-regulation for *TA11145*. Auto-regulation of this gene was supported by demonstration that the encoded AP2 domain binds specifically to a probe representing a double (A)CACAC(A) type motif present in the upstream IGR. Multiple auto-regulatory sites were also reported for *P*. *falciparum AP2-G* [13]. One possibility is that these sites generate and/or detect a gradient of DNA binding factor that influences when a commitment event will occur. It is known that double motifs allow higher interaction affinities and slower dissociation of DNA binding proteins [51].

Multiple domains that recognise (A)CACAC(A) type motifs allow speculation that different AP2 factors could bind to the same promoter and potentially compete for binding if co-expressed. Indeed, the TA11145 AP2 domain can bind to the motif predicted for the TA07100 domain (S10 Fig). In addition, the data of Campbell *et al*. [9] indicate that individual domains bind to variants but show greater affinity to their preferred motif. The expression patterns of genes in *T*. *annulata* encoding AP2 domains that are predicted to bind (A)CACAC(A) motifs overlap during differentiation to the merozoite (see Fig 2), with TA11145 showing significant up-regulation relative to TA07100. These findings support the previous stochastic model of differentiation for *T*. *annulata* [2], where a functional overlap between regulatory factors of different life-cycle stages was predicted. In an update of this model, we propose that low-level expression of merozoite genes involves regulation by macroschizont (AP2) factors that bind to (A)CACAC(A) motifs in the upstream region of *TA11145*. Thus expression of *TA11145* at the macroschizont stage may be influenced by a stoichiometric relationship between competing factors that bind to (A)CACAC(A) motifs and promote low (repressed) or elevated (activated) gene expression. Following placement at 41°C, an elevation in protein levels relative to DNA template occurs and generates a skewed increase in *TA11145* expression over time via an auto-regulatory loop. This loop would be promoted by preferential binding of the AP2 domain encoded by *TA11145* to multiple (A)CACAC(A) sites in its own upstream region. One prediction of the model is that the relative level of competing factors would differ between parasite lines attenuated or competent for a stage-differentiation event. This appears to be the case for genes encoding AP2 domains that bind the (A)CACAC(A) motif, with a significant increase in the level of *TA11145* expression, relative to *TA07100*, in an infected cell line that is able to undergo differentiation to the merozoite compared to a line which has lost this ability (Fig 7).

Further experimental data are required to validate, refute or modify the above model. Nevertheless, it could account for a number of findings common across stage-differentiation events of different Apicomplexan genera. These include, low level expression of genes in the life-cycle prior to the stage where they are expressed at a high level [4,12–14]; a gene expression profile that is intermediate between two stages that may be reversed or progressed, depending on culture conditions [3,4,16,52,53]; evidence for multiple DNA-binding proteins that bind to related motifs and show a temporal order of expression linked to stage-differentiation [9,11,12]; and parasite lines with marked, quantitative differences in their potential to undergo a stage-differentiation event [4,11,13,42,54]. It should be noted though that even if a common mechanism operates across genera, it is unlikely that the target genes regulated during stage differentiation steps will be necessarily conserved.

Recognition of closely related binding motifs by multiple DNA binding proteins shared across genera operates to regulate developmental gene expression in higher eukaryotes, with auto-regulation and competition for binding sites evident [55]. For example, the double GATA motif upstream of the GATA-1 gene that is required for developmental expression is first bound by GATA-2 to initiate expression of GATA-1, followed by preferential GATA-1 binding and auto-regulation via the same motif [51,56,57]. Thus, we propose that competition between related DNA binding proteins can determine whether an Apicomplexan parasite stays at the same life-cycle stage or progresses to the next, and may be a remnant of an ancestral stochastic mechanism of cellular differentiation retained in both lower and higher eukaryotes.

## Supporting Information

S1 Table List of biotinylated oligonucleotide probes used in EMSA. Motifs predicted for *T*. *annulata* AP2 binding domains are in bold, mutated motifs are underlined.
(PDF)

S2 Table List of top 100 genes displaying elevated expression level from macroschizont (Day 0) to merozoite stage (Day 9) identified by RP analysis of microarray data (FDR < 0.05). (PDF)

S3 Table List of top 100 genes displaying reduced expression levels from macroschizont (Day 0) to merozoite stage (Day 9) identified by RP analysis of microarray data (FDR < 0.05). (PDF)

S4 Table Differential expression of TaApiAP2 genes between infected cell lines, D7 versus the D7B12 line that is attenuated for differentiation to the merozoite. Differential expression between cell lines was identified by RP analysis of microarray data (FDR < 0.05). Genes encoding AP2 domains predicted to bind (A)CACAC(A) motifs are highlighted with a blue bar.
(PDF)

S1 Fig QRT-PCR analysis of TA15705 gene expression, plotted as fold change in expression relative to the Day 0 (macroschizont) at Day 4, Day 7 and Day 9 (merozoite), and piroplasm stage; * significant difference (P value ≤ 0.05) relative to Day 0 (macroschizont stage). (PDF)

S2 Fig Alignment of ApiAP2 domains of the four genes of *T*. *annulata* up-regulated during differentiation to the merozoite: significant divergence across the paralogues is apparent. Regions of predicted secondary structure are indicated above the alignment and were predicted with Phyre^2^ using three independent secondary structure prediction programs: Psi-Pred [58], SSPro [59] and JNet [60]. * identity,:. similarity.
(PDF)

S3 Fig Correlation plots of expression of ApiAP2 domain-encoding genes and putative target genes. ApiAP2 gene expression profile is shown in red, while the average profile of putative target genes possessing the motif bound by the *P*. *falciparum* orthologous domain are shown in blue, a significant Pearson correlation coefficient value is indicated for each plot.
(PDF)

S4 Fig EMSA performed with 0.7 μg of purified GST-TA16485D (TaAP2.me3) and 20 fmol of biotin-labelled double stranded oligo probe containing the core TCTATA motif bound by the orthologous domain (PF3D7_1239200) in *P*. *falciparum*: lane 1, probe only; lane 2, probe + GST-TA16485D; lane 3, probe + GST-TA16485D + cold competitor (2 pmol); lane 4, probe + GST-TA16485D + competitor (4 pmol). (PDF)

S5 Fig EMSA performed with GST-TA12015D (TaAP2.me2) fusion protein and 20 fmol of biotin-labelled probe (ATTGTTAATTCCCCATCCAGATCTATAAAA) representing the core motif TCCCCAT (C-box/G-box): lane 1, probe only; lane 2, 0.6 g of fusion protein + probe; lane 3, 0.9 μg of fusion protein + probe; lane 4, 1.2 μg of fusion protein + probe; lane 5, 1.6 μg of fusion protein + probe. (PDF)

S6 Fig EMSA performed with probe representing the double (A)CACAC(A) motif upstream of gene TA11145: lane 1, probe only; lane 2, probe + nuclear extract generated from uninfected BL20 cells; lane 3, probe + PNE from D7 culture Day 9 of differentiation to merozoite time-course. Letters denote shift positions detected in both infected and uninfected cells at Day 0 (37°C), shift B was only detected in extracts derived from infected cells undergoing merogony (Day 9, 41°C).
(PDF)

S7 Fig Intergenic region upstream of the protein-encoding region of TA11145: (A)CACAC(A) motifs are highlighted in red, motifs spatially conserved in the IGR of the *T*. *parva* orthologue (TP04_0872) are in bold; the probe used for the EMSA is underlined. (PDF)

S8 Fig Alignment of ApiAP2 domain encoded *by TA07100* with orthologous domains identified by BLAST analysis from *T*. *parva*, *T*. *orientalis*, *B*. *bovis*, and *P*. *falciparum*: strong conservation of the domain across the related species (98% and 100% identity) and genera (91% identity, 96% similarity with *B*. *bovis*; 78% identity, 91% similarity with *P*. *falciparum*) is apparent. The domain in *P*. *falciparum* has been shown to bind an (A)CACAC(A) type motif. Regions of predicted secondary structure are indicated above the alignment and were predicted with Phyre^2^ using three independent secondary structure prediction programs: Psi-Pred [58], SSPro [59] and JNet [60]. * identity,:. similarity.
(PDF)

S9 Fig QRT-PCR data plotted as fold-change in elevated expression (log_2_) for ApiAP2 domain encoding gene *TA13515* (TaAP2.g) in the differentiation competent D7 cell line versus the attenuated D7B12 cell line. Fold-change in expression between cell lines was computed at Day 0 (macroschizont) and Day 4 and Day 7 points of a time-course of differentiation to the merozoite; * denotes significant (P value ≤ 0.05) fold-change elevated expression in D7 vs D7B12.
(PDF)

S10 Fig EMSA performed with GST-AP2 fusion proteins representing TA11145, TA13515, TA12015 and TA16485 domains and a probe representing the CACACAC core motif bound by the *P*. *falciparum* orthologue (PF3D7_0420300 (PFD0985w.D1) of the TA07100 domain: lane 1, probe alone; lane 2, 0.7 μg GST-TA11145D fusion protein + probe; lane 3, 0.7 μg GST-TA13515D fusion protein + probe; lane 4, 0.7 μg GST-TA12015D fusion protein + probe; lane 5, 0.7 μg GST-TA16485D fusion protein + probe; lane 6, 0.7 μg GST-TA11145D fusion protein + probe representing the core motif TGCATGCA bound by the *P*. *falciparum* domain of PF3D7_1466400 (PF14_0633). Arrow denotes the shift position obtained with GST-TA11145D, the more minor shift obtained with GST-TA13515D may be indicative weaker/partial recognition of the probe.
(PDF)

## References

[1] Dyer M, Day KP (2003) Regulation of the rate of asexual growth and commitment to sexual development by diffusible factors from *in vitro* cultures of *Plasmodium falciparum* . Am J Trop Med Hyg 68: 403–409. 12875287

[2] Shiels BR (1999) Should I stay or should I go now? A stochastic model of stage differentiation in *Theileria annulata* . ParasitolToday 15: 241–245. 10.1016/s0169-4758(99)01451-9 10366832

[3] Reininger L, Garcia M, Tomlins A, Müller S, Doerig C (2012) The *Plasmodium falciparum*, Nima-related kinase Pfnek-4: a marker for asexual parasites committed to sexual differentiation. Malaria journal 11: 1–11. 22849771 10.1186/1475-2875-11-250 PMC3495404

[4] Shiels BR, Smyth A, Dickson J, McKellar S, Tetley L, et al (1994) A stoichiometric model of stage differentiation in the protozoan parasite *Theileria annulata* . MolCell Differ 2 101–125.

[5] Shiels B, Swan D, McKellar S, Aslam N, Dando C, et al (1998) Directing differentiation in *Theileria annulata*: old methods and new possibilities for control of apicomplexan parasites. IntJParasitol 28: 1659–1670. 10.1016/s0020-7519(98)00131-3 9846602

[6] Loose M, Swiers G, Patient R (2007) Transcriptional networks regulating hematopoietic cell fate decisions. Curr Opin Hematol 14: 307–314. 17534154 10.1097/MOH.0b013e3281900eee

[7] Balaji S, Babu MM, Iyer LM, Aravind L (2005) Discovery of the principal specific transcription factors of Apicomplexa and their implication for the evolution of the AP2-integrase DNA binding domains. Nucleic Acids Res 33: 3994–4006. 16040597 10.1093/nar/gki709 PMC1178005

[8] Jofuku KD, Den Boer B, Van Montagu M, Okamuro JK (1994) Control of Arabidopsis flower and seed development by the homeotic gene APETALA2. The Plant Cell Online 6: 1211–1225. 10.1105/tpc.6.9.1211 PMC160514 7919989

[9] Campbell TL, De Silva EK, Olszewski KL, Elemento O, Llinás M (2010) Identification and genome-wide prediction of DNA binding specificities for the ApiAP2 family of regulators from the malaria parasite. PLoS Pathog 6: e1001165 10.1371/journal.ppat.1001165 21060817 PMC2965767

[10] Yuda M, Iwanaga S, Shigenobu S, Kato T, Kaneko I (2010) Transcription factor AP2‐Sp and its target genes in malarial sporozoites. Molecular microbiology 75: 854–863. 10.1111/j.1365-2958.2009.07005.x 20025671

[11] Radke JB, Lucas O, De Silva EK, Ma Y, Sullivan WJ, et al (2013) ApiAP2 transcription factor restricts development of the Toxoplasma tissue cyst. Proceedings of the National Academy of Sciences 110: 6871–6876. 10.1073/pnas.1300059110 PMC3637731 23572590

[12] Walker R, Gissot M, Croken MM, Huot L, Hot D, et al (2013) The Toxoplasma nuclear factor TgAP2XI‐4 controls bradyzoite gene expression and cyst formation. Molecular microbiology 87: 641–655. 10.1111/mmi.12121 23240624 PMC3556193

[13] Kafsack BF, Rovira-Graells N, Clark TG, Bancells C, Crowley VM, et al (2014) A transcriptional switch underlies commitment to sexual development in malaria parasites. Nature 507: 248–252. 10.1038/nature12920 24572369 PMC4040541

[14] Sinha A, Hughes KR, Modrzynska KK, Otto TD, Pfander C, et al (2014) A cascade of DNA-binding proteins for sexual commitment and development in Plasmodium. Nature 507: 253–257. 10.1038/nature12970 24572359 PMC4105895

[15] Mhadhbi M, Naouach A, Boumiza A, Chaabani MF, Ben Abderazzak S, et al (2010) *In vivo* evidence for the resistance of *Theileria annulata* to buparvaquone. VetParasitol 169: 241–247. 10.1016/j.vetpar.2010.01.013 20185242

[16] Shiels B, Aslam N, McKellar S, Smyth A, Kinnaird J (1997) Modulation of protein synthesis relative to DNA synthesis alters the timing of differentiation in the protozoan parasite *Theileria annulata* . Journal of cell science 110: 1441–1451. 9224762 10.1242/jcs.110.13.1441

[17] Shiels B, Fox M, McKellar S, Kinnaird J, Swan D (2000) An upstream element of the TamS1 gene is a site of DNA-protein interactions during differentiation to the merozoite in *Theileria annulata* . JCell Sci 113 (Pt 12): 2243–2252. 10825296 10.1242/jcs.113.12.2243

[18] Olobo JO, Black SJ (1989) Selected phenotypic and cloning properties of a bovine lymphoblastoid cell line, BL20. VetImmunolImmunopathol 20: 165–172. 10.1016/0165-2427(89)90096-2 2650460

[19] Kinnaird JH, Weir W, Durrani Z, Pillai SS, Baird M, et al (2013) A Bovine Lymphosarcoma Cell Line Infected with *Theileria annulata* Exhibits an Irreversible Reconfiguration of Host Cell Gene Expression. PLoS One 8: e66833 23840536 10.1371/journal.pone.0066833 PMC3694138

[20] Williamson S, Tait A, Brown D, Walker A, Beck P, et al (1989) *Theileria annulata* sporozoite surface antigen expressed in *Escherichia coli* elicits neutralizing antibody. ProcNatlAcadSciUSA 86: 4639–4643. 10.1073/pnas.86.12.4639 PMC287326 2499888

[21] Pain A, Renauld H, Berriman M, Murphy L, Yeats CA, et al (2005) Genome of the host-cell transforming parasite *Theileria annulata* compared with *T*. *parva* . Science 309: 131–133. 15994557 10.1126/science.1110418

[22] Kent WJ (2002) BLAT—the BLAST-like alignment tool. Genome Res 12: 656–664. 11932250 10.1101/gr.229202 PMC187518

[23] Roche FM, Hokamp K, Acab M, Babiuk LA, Hancock RE, et al (2004) ProbeLynx: a tool for updating the association of microarray probes to genes. Nucleic Acids Res 32: W471–W474. 15215432 10.1093/nar/gkh452 PMC441590

[24] Kane MD, Jatkoe TA, Stumpf CR, Lu J, Thomas JD, et al (2000) Assessment of the sensitivity and specificity of oligonucleotide (50mer) microarrays. Nucleic Acids Res 28: 4552–4557. 11071945 10.1093/nar/28.22.4552 PMC113865

[25] Irizarry RA, Hobbs B, Collin F, Beazer-Barclay YD, Antonellis KJ, et al (2003) Exploration, normalization, and summaries of high density oligonucleotide array probe level data. Biostatistics 4: 249–264. 12925520 10.1093/biostatistics/4.2.249

[26] Edgar R, Domrachev M, Lash AE (2002) Gene Expression Omnibus: NCBI gene expression and hybridization array data repository. Nucleic Acids Res 30: 207–210. 11752295 10.1093/nar/30.1.207 PMC99122

[27] Breitling R, Armengaud P, Amtmann A, Herzyk P (2004) Rank products: a simple, yet powerful, new method to detect differentially regulated genes in replicated microarray experiments. FEBS Lett 573: 83–92. 15327980 10.1016/j.febslet.2004.07.055

[28] Benjamini Y, Hochberg Y (1995) Controlling the False Discovery Rate: A Practical and Powerful Approach to Multiple Testing. Journal of the Royal Statistical Society Series B (Methodological) 57 289–300.

[29] Mason PJ, Shiels BR, Tait A, Beck P, Hall R (1989) Sequence and expression of a gene from *Theileria annulata* coding for a 70-kilodalton heat-shock protein. Mol Biochem Parasitol 37: 27–35. 2515435 10.1016/0166-6851(89)90099-6

[30] Livak KJ, Schmittgen TD (2001) Analysis of Relative Gene Expression Data Using Real-Time Quantitative PCR and the 2− ΔΔCT Method. Methods 25: 402–408. 11846609 10.1006/meth.2001.1262

[31] Guo X, Silva JC (2008) Properties of non-coding DNA and identification of putative cis-regulatory elements in *Theileria parva* . BMCGenomics 9: 582. 10.1186/1471-2164-9-582 PMC2612703 19055776

[32] Bailey TL, Williams N, Misleh C, Li WW (2006) MEME: discovering and analyzing DNA and protein sequence motifs. Nucleic Acids Res 34: W369–W373. 16845028 10.1093/nar/gkl198 PMC1538909

[33] Oberstaller J, Joseph SJ, Kissinger JC (2013) Genome-wide upstream motif analysis of *Cryptosporidium parvum* genes clustered by expression profile. BMC Genomics 14: 516 10.1186/1471-2164-14-516 23895416 PMC3734150

[34] Stamatakis A (2014) RAxML version 8: a tool for phylogenetic analysis and post-analysis of large phylogenies. Bioinformatics 30: 1312–1313. 10.1093/bioinformatics/btu033 24451623 PMC3998144

[35] Schmuckli-Maurer J, Casanova C, Schmied S, Affentranger S, Parvanova I, et al (2009) Expression analysis of the *Theileria parva* subtelomere-encoded variable secreted protein gene family. PLoSONE 4: e4839. 10.1371/journal.pone.0004839 PMC2657828 19325907

[36] Swan DG, Stern R, McKellar S, Phillips K, Oura CA, et al (2001) Characterisation of a cluster of genes encoding *Theileria annulata* AT hook DNA-binding proteins and evidence for localisation to the host cell nucleus. JCell Sci 114: 2747–2754. 11683409 10.1242/jcs.114.15.2747

[37] Weir W, Karagenc T, Baird M, Tait A, Shiels BR (2010) Evolution and diversity of secretome genes in the apicomplexan parasite *Theileria annulata* . BMCGenomics 11: 42. 10.1186/1471-2164-11-42 PMC2826314 20082698

[38] MacHugh ND, Weir W, Burrells A, Lizundia R, Graham SP, et al (2011) Extensive polymorphism and evidence of immune selection in a highly dominant antigen recognised by bovine CD8 T cells specific for *Theileria annulata* . InfectImmun. 10.1128/IAI.01285-10 PMC3088144 21300773

[39] Oberstaller J, Pumpalova Y, Schieler A, Llinás M, Kissinger JC (2014) The *Cryptosporidium parvum* ApiAP2 gene family: insights into the evolution of apicomplexan AP2 regulatory systems. Nucleic Acids Res: gku 500. 10.1093/nar/gku500 PMC4117751 24957599

[40] De Silva EK, Gehrke AR, Olszewski K, León I, Chahal JS, et al (2008) Specific DNA-binding by apicomplexan AP2 transcription factors. Proceedings of the National Academy of Sciences 105: 8393–8398. 10.1073/pnas.0801993105 PMC2423414 18541913

[41] Pieszko M (2015) Molecular regulation of the macroschizont to merozoite stage differentiation in *Theileria annulata*: University of Glasgow.

[42] Skariah S, McIntyre MK, Mordue DG (2010) *Toxoplasma gondii*: determinants of tachyzoite to bradyzoite conversion. Parasitol Res 107: 253–260. 10.1007/s00436-010-1899-6 20514494 PMC3327608

[43] Shiels B, Langsley G, Weir W, Pain A, McKellar S, et al (2006) Alteration of host cell phenotype by *Theileria annulata* and *Theileria parva*: mining for manipulators in the parasite genomes. IntJParasitol 36: 9–21. 10.1016/j.ijpara.2005.09.002 16221473

[44] Bishop R, Shah T, Pelle R, Hoyle D, Pearson T, et al (2005) Analysis of the transcriptome of the protozoan *Theileria parva* using MPSS reveals that the majority of genes are transcriptionally active in the schizont stage. Nucleic Acids Res 33: 5503–5511. 16186131 10.1093/nar/gki818 PMC1236717

[45] Behnke MS, Wootton JC, Lehmann MM, Radke JB, Lucas O, et al (2010) Coordinated progression through two subtranscriptomes underlies the tachyzoite cycle of *Toxoplasma gondii* . PLoS One 5: e12354 10.1371/journal.pone.0012354 20865045 PMC2928733

[46] Mehlhorn H, Schein E (1984) The piroplasms: life cycle and sexual stages. AdvParasitol 23: 37–103. 10.1016/s0065-308x(08)60285-7 6442536

[47] Ankarklev J, Brancucci N, Goldowitz I, Mantel P-Y, Marti M (2014) Sex: How Malaria Parasites Get Turned On. Current Biology 24: R368–R370. 10.1016/j.cub.2014.03.046 24801188

[48] Chaubey S, Grover M, Tatu U (2014) Endoplasmic reticulum stress triggers gametocytogenesis in the malaria parasite. Journal of Biological Chemistry 289: 16662–16674. 10.1074/jbc.M114.551549 24755215 PMC4059112

[49] Bohne W, Wirsing A, Gross U (1997) Bradyzoite-specific gene expression in *Toxoplasma gondii* requires minimal genomic elements. Mol Biochem Parasitol 85: 89–98. 9108551 10.1016/s0166-6851(96)02814-9

[50] Young JA, Johnson JR, Benner C, Yan SF, Chen K, et al (2008) In silico discovery of transcription regulatory elements in *Plasmodium falciparum* . BMC Genomics 9: 70 10.1186/1471-2164-9-70 18257930 PMC2268928

[51] Ghirlando R, Trainor CD (2003) Determinants of GATA-1 Binding to DNA: the role of non-finger residues. Journal of Biological Chemistry 278: 45620–45628. 12941967 10.1074/jbc.M306410200

[52] Radke JR, Behnke MS, Mackey AJ, Radke JB, Roos DS, et al (2005) The transcriptome of *Toxoplasma gondii* . BMC biology 3: 26 16324218 10.1186/1741-7007-3-26 PMC1325263

[53] Radke JR, Guerini MN, Jerome M, White MW (2003) A change in the premitotic period of the cell cycle is associated with bradyzoite differentiation in *Toxoplasma gondii* . Mol Biochem Parasitol 131: 119–127. 14511810 10.1016/s0166-6851(03)00198-1

[54] Jeffers T (1975) Attenuation of *Eimeria tenella* through selection for precociousness. The Journal of parasitology: 1083–1090. 1195070

[55] Hueber SD, Lohmann I (2008) Shaping segments: Hox gene function in the genomic age. Bioessays 30: 965–979. 10.1002/bies.20823 18798525

[56] Ohneda K, Yamamoto M (2002) Roles of hematopoietic transcription factors GATA-1 and GATA-2 in the development of red blood cell lineage. Acta haematologica 108: 237–245. 12432220 10.1159/000065660

[57] Vyas P, McDevitt MA, Cantor AB, Katz SG, Fujiwara Y, et al (1999) Different sequence requirements for expression in erythroid and megakaryocytic cells within a regulatory element upstream of the GATA-1 gene. Development 126: 2799–2811. 10331989 10.1242/dev.126.12.2799

[58] Jones DT (1999) Protein secondary structure prediction based on position-specific scoring matrices. Journal of molecular biology 292: 195–202. 10493868 10.1006/jmbi.1999.3091

[59] Magnan CN, Baldi P (2014) SSpro/ACCpro 5: almost perfect prediction of protein secondary structure and relative solvent accessibility using profiles, machine learning and structural similarity. Bioinformatics 30: 2592–2597. 10.1093/bioinformatics/btu352 24860169 PMC4215083

[60] Cole C, Barber JD, Barton GJ (2008) The Jpred 3 secondary structure prediction server. Nucleic Acids Res 36: W197–W201. 10.1093/nar/gkn238 18463136 PMC2447793

