# Supplementary material for: ApiAP2 Factors as Candidate Regulators of Stochastic Commitment to Merozoite Production in Theileria annulata
Source: PLoS Negl Trop Dis. 2015 Aug 14;9(8):e0003933. doi: 10.1371/journal.pntd.0003933 (PMC4537280; doi:10.1371/journal.pntd.0003933)
Supplement: S1 Table — Motifs predicted for T. annulata AP2 binding domains are in bold, mutated motifs are underlined. (PDF) [file pntd.0003933.s001.pdf]

S1 Table: List of biotinylated oligonucleotide probes used in EMSA

Probe name	Sequence 5'->3'	Description
**Bio-2x(A)CACAC(A)**	Bio-GAT**ACACAC**TTATG**CACACA**CA	Target double motif for AP2 domain encoded by TA11145 from upstream region of TA11145
**Bio(A)CACAC(A)-MUT**	Bio-GATATAGAATTATGCAGAATAT	Mutated double motif from upstream region of TA11145
**Bio-GTGTAC**	Bio-AATATTATAATAGTCGTAGCCATCAAT**GTGTACAC**ATGGTAATATAGATTTTCGTTTATATT	Target motif for AP2 domain of TA13515; motif for PBANKA_143750/PFL1085w
**Bio-GTGTAC-MUT**	Bio-AATATTATAATAGTCGTAGCCATCAATATATAAAAATGGTAATATAGATTTTCGTTTATATT	Mutated target motif for AP2 domain encoded by TA13515
**Bio-TCTATA**	Bio-ATTGTTAATTCCCCATCCAGA**TCTATA**AAA	Target TCTATA core motif for the AP2 domain encoded by TA16485.
