# Supplementary material for: ApiAP2 Factors as Candidate Regulators of Stochastic Commitment to Merozoite Production in Theileria annulata
Source: PLoS Negl Trop Dis. 2015 Aug 14;9(8):e0003933. doi: 10.1371/journal.pntd.0003933 (PMC4537280; doi:10.1371/journal.pntd.0003933)
Supplement: S2 Table — (PDF) [file pntd.0003933.s002.pdf]

## S2 Table: Top 100 genes displaying elevated expression, macroschizont (Day 0) to merozoite stage (Day 9)

| Gene ID | Annotation | FC | RP score | EE | FDR |
|---|---|---|---|---|---|
| **TA05870** | rhoptry-associated protein, putative | 5.12 | $3.13 \times 10^{-31}$ | 0.00 | 0.0000 |
| **TA14665** | hypothetical protein | 5.13 | $1.41 \times 10^{-30}$ | 0.00 | 0.0000 |
| **TA08360** | hypothetical protein, conserved | 4.64 | $1.69 \times 10^{-28}$ | 0.00 | 0.0000 |
| **TA21080** | Map2 kinase, putative | 4.16 | $2.40 \times 10^{-26}$ | 0.00 | 0.0000 |
| **TA05340** | hypothetical protein, conserved | 4.03 | $1.21 \times 10^{-25}$ | 0.00 | 0.0000 |
| **TA16660** | hypothetical protein, conserved | 3.96 | $2.36 \times 10^{-25}$ | 0.00 | 0.0000 |
| **TA05495** | hypothetical protein | 3.89 | $2.65 \times 10^{-25}$ | 0.00 | 0.0000 |
| **TA13045** | hypothetical protein, conserved | 3.67 | $5.49 \times 10^{-24}$ | 0.00 | 0.0000 |
| **TA13825** | hypothetical protein | 3.63 | $1.28 \times 10^{-23}$ | 0.00 | 0.0000 |
| **TA07585** | hypothetical protein | 3.51 | $2.58 \times 10^{-23}$ | 0.00 | 0.0000 |
| **TA19390** | hypothetical protein, conserved | 3.40 | $1.11 \times 10^{-22}$ | 0.00 | 0.0000 |
| **TA18005** | hypothetical protein | 3.38 | $2.06 \times 10^{-22}$ | 0.00 | 0.0000 |
| **TA19040** | hypothetical protein, conserved | 3.35 | $2.30 \times 10^{-22}$ | 0.00 | 0.0000 |
| **TA19445** | hypothetical protein, conserved | 3.24 | $8.27 \times 10^{-22}$ | 0.00 | 0.0000 |
| **TA11905** | hypothetical protein | 3.43 | $9.64 \times 10^{-22}$ | 0.00 | 0.0000 |
| **TA20020** | hypothetical protein, conserved | 3.08 | $4.25 \times 10^{-21}$ | 0.02 | 0.0013 |
| **TA14680** | hypothetical protein | 3.02 | $9.70 \times 10^{-21}$ | 0.03 | 0.0018 |
| **TA21400** | hypothetical protein | 2.93 | $2.34 \times 10^{-20}$ | 0.04 | 0.0022 |
| **TA17325** | integral membrane protein, putative | 2.95 | $2.41 \times 10^{-20}$ | 0.04 | 0.0021 |
| **TA16375** | hypothetical protein, conserved | 2.94 | $3.47 \times 10^{-20}$ | 0.04 | 0.0020 |
| **TA05760** | rhoptry-associated protein, putative | 2.91 | $3.70 \times 10^{-20}$ | 0.05 | 0.0024 |
| **TA21395** | hypothetical protein | 2.95 | $4.08 \times 10^{-20}$ | 0.05 | 0.0023 |
| **TA04105** | cysteine proteinase, putative | 2.90 | $5.75 \times 10^{-20}$ | 0.07 | 0.0030 |
| **TA13515** | hypothetical protein, conserved | 2.88 | $8.26 \times 10^{-20}$ | 0.07 | 0.0029 |
| **TA14955** | hypothetical protein | 2.85 | $1.43 \times 10^{-19}$ | 0.08 | 0.0032 |
| **TA13215** | hypothetical protein, conserved | 2.79 | $1.43 \times 10^{-19}$ | 0.08 | 0.0031 |

| | | | | | |
|---|---|---|---|---|---|
| **TA16485** | hypothetical protein, conserved | 2.78 | $2.13 \times 10^{-19}$ | 0.09 | 0.0033 |
| **TA11455** | hypothetical protein, conserved | 2.76 | $2.77 \times 10^{-19}$ | 0.09 | 0.0032 |
| **TA18855** | SfiI-subtelomeric fragment related protein family member, putative | 2.79 | $3.15 \times 10^{-19}$ | 0.10 | 0.0035 |
| **TA16420** | hypothetical protein | 2.83 | $3.28 \times 10^{-19}$ | 0.10 | 0.0033 |
| **TA18195** | hypothetical protein | 2.75 | $4.13 \times 10^{-19}$ | 0.12 | 0.0039 |
| **TA15485** | hypothetical protein, conserved | 2.74 | $4.60 \times 10^{-19}$ | 0.13 | 0.0041 |
| **TA14205** | hypothetical protein | 2.67 | $9.74 \times 10^{-19}$ | 0.16 | 0.0049 |
| **TA16155** | hypothetical protein | 2.66 | $1.04 \times 10^{-18}$ | 0.16 | 0.0047 |
| **TA17100** | hypothetical protein | 2.67 | $1.20 \times 10^{-18}$ | 0.17 | 0.0049 |
| **TA04660** | hypothetical protein, conserved | 2.64 | $1.28 \times 10^{-18}$ | 0.17 | 0.0047 |
| **TA21390** | Theileria parva Tpr-related protein, putative | 2.65 | $1.29 \times 10^{-18}$ | 0.17 | 0.0046 |
| **TA11285** | hypothetical protein | 2.70 | $1.62 \times 10^{-18}$ | 0.19 | 0.0050 |
| **TA17490** | | 2.65 | $2.07 \times 10^{-18}$ | 0.19 | 0.0049 |
| **TA08480** | hypothetical protein, conserved | 2.61 | $2.91 \times 10^{-18}$ | 0.21 | 0.0053 |
| **TA07985** | hypothetical protein | 2.61 | $4.77 \times 10^{-18}$ | 0.22 | 0.0054 |
| **TA07025** | Tpr-related protein family member, putative | 2.56 | $5.58 \times 10^{-18}$ | 0.23 | 0.0055 |
| **TA21385** | hypothetical protein | 2.54 | $5.62 \times 10^{-18}$ | 0.23 | 0.0054 |
| **TA03260** | hypothetical protein | 2.53 | $6.10 \times 10^{-18}$ | 0.23 | 0.0052 |
| **TA20555** | myosin a, putative | 2.49 | $1.09 \times 10^{-17}$ | 0.30 | 0.0067 |
| **TA19610** | hypothetical protein, conserved | 2.49 | $1.91 \times 10^{-17}$ | 0.34 | 0.0074 |
| **TA14210** | hypothetical protein | 2.44 | $1.92 \times 10^{-17}$ | 0.34 | 0.0072 |
| **TA07920** | hypothetical protein | 2.46 | $2.96 \times 10^{-17}$ | 0.43 | 0.0090 |
| **TA15445** | Tpr-related protein family member, putative | 2.40 | $3.39 \times 10^{-17}$ | 0.48 | 0.0098 |
| **TA15620** | hypothetical protein | 2.40 | $4.06 \times 10^{-17}$ | 0.52 | 0.0104 |
| **TA15625** | hypothetical protein | 2.41 | $4.21 \times 10^{-17}$ | 0.52 | 0.0102 |
| **TA19275** | hypothetical protein | 2.42 | $4.43 \times 10^{-17}$ | 0.53 | 0.0102 |
| **TA14285** | SfiI-subtelomeric fragment related protein family member, putative | 2.41 | $4.53 \times 10^{-17}$ | 0.53 | 0.0100 |

| | | | | | |
|---|---|---|---|---|---|
| **TA07630** | hypothetical protein, conserved | 2.39 | $5.90 \times 10^{-17}$ | 0.56 | 0.0104 |
| **TA16685** | polymorphic antigen precursor-like protein, putative | 2.39 | $6.37 \times 10^{-17}$ | 0.56 | 0.0102 |
| **TA13890** | hypothetical protein | 2.32 | $2.07 \times 10^{-16}$ | 0.83 | 0.0148 |
| **TA04565** | hypothetical protein | 2.30 | $2.20 \times 10^{-16}$ | 0.84 | 0.0147 |
| **TA19505** | hypothetical protein, conserved | 2.30 | $2.37 \times 10^{-16}$ | 0.84 | 0.0145 |
| **TA17055** | hypothetical protein | 2.29 | $2.42 \times 10^{-16}$ | 0.84 | 0.0142 |
| **TA08235** | hypothetical protein | 2.30 | $2.42 \times 10^{-16}$ | 0.84 | 0.0140 |
| **TA14310** | hypothetical protein | 2.28 | $3.29 \times 10^{-16}$ | 1.00 | 0.0164 |
| **TA13540** | hypothetical protein | 2.27 | $3.34 \times 10^{-16}$ | 1.00 | 0.0161 |
| **TA17358** | | 2.25 | $3.82 \times 10^{-16}$ | 1.01 | 0.0160 |
| **TA13530** | phosphate transporter, putative | 2.25 | $3.97 \times 10^{-16}$ | 1.01 | 0.0158 |
| **TA15490** | hypothetical protein | 2.25 | $4.84 \times 10^{-16}$ | 1.04 | 0.0160 |
| **TA17115** | SfiI-subtelomeric fragment related protein family member, putative | 2.25 | $5.20 \times 10^{-16}$ | 1.07 | 0.0162 |
| **TA20435** | hypothetical protein | 2.23 | $5.44 \times 10^{-16}$ | 1.12 | 0.0167 |
| **TA06795** | hypothetical protein, conserved | 2.23 | $5.55 \times 10^{-16}$ | 1.14 | 0.0168 |
| **TA18275** | hypothetical protein | 2.22 | $5.91 \times 10^{-16}$ | 1.17 | 0.0170 |
| **TA20150** | Theileria-specific hypothetical protein | 2.22 | $6.00 \times 10^{-16}$ | 1.17 | 0.0167 |
| **TA14130** | Tpr-related protein family member, putative | 2.23 | $6.31 \times 10^{-16}$ | 1.19 | 0.0168 |
| **TA04355** | hypothetical protein | 2.19 | $8.91 \times 10^{-16}$ | 1.31 | 0.0182 |
| **TA13940** | hypothetical protein | 2.21 | $9.32 \times 10^{-16}$ | 1.36 | 0.0186 |
| **TA07435** | SfiI-subtelomeric fragment related protein family member, putative | 2.15 | $1.15 \times 10^{-15}$ | 1.47 | 0.0199 |
| **TA10645** | hypothetical protein | 2.18 | $1.20 \times 10^{-15}$ | 1.49 | 0.0199 |
| **TA20985** | hypothetical protein | 2.15 | $1.30 \times 10^{-15}$ | 1.55 | 0.0204 |
| **TA15095** | Tpr-related protein family member, putative | 2.18 | $1.35 \times 10^{-15}$ | 1.55 | 0.0201 |
| **TA15090** | Tpr-related protein family member, putative | 2.17 | $1.37 \times 10^{-15}$ | 1.55 | 0.0199 |
| **TA15355** | hypothetical protein | 2.15 | $1.51 \times 10^{-15}$ | 1.62 | 0.0205 |

| | | | | | |
|---|---|---|---|---|---|
| **TA19675** | hypothetical protein | 2.15 | $1.58 \times 10^{-15}$ | 1.65 | 0.0206 |
| **TA17960** | hypothetical protein | 2.15 | $1.62 \times 10^{-15}$ | 1.65 | 0.0204 |
| **TA13535** | hypothetical protein | 2.11 | $1.62 \times 10^{-15}$ | 1.65 | 0.0201 |
| **TA05375** | hypothetical protein | 2.16 | $1.67 \times 10^{-15}$ | 1.71 | 0.0206 |
| **TA14335** | hypothetical protein | 2.15 | $1.72 \times 10^{-15}$ | 1.73 | 0.0206 |
| **TA10690** | ubiquitin-conjugating enzyme E2. putative | 2.12 | $2.36 \times 10^{-15}$ | 1.88 | 0.0221 |
| **TA19975** | integral membrane protein, putative | 2.11 | $2.73 \times 10^{-15}$ | 1.95 | 0.0227 |
| **TA03850** | Tpr-related protein family member, putative | 2.13 | $2.85 \times 10^{-15}$ | 1.98 | 0.0228 |
| **TA14120** | Tpr-related protein family member, putative | 2.09 | $3.18 \times 10^{-15}$ | 2.04 | 0.0232 |
| **TA03540** | hypothetical protein | 2.06 | $3.40 \times 10^{-15}$ | 2.12 | 0.0238 |
| **TA14135** | Tpr-related protein family member, putative | 2.10 | $3.87 \times 10^{-15}$ | 2.15 | 0.0239 |
| **TA03755** | sporozoite surface antigen (spag -1) | 2.08 | $3.87 \times 10^{-15}$ | 2.15 | 0.0236 |
| **TA17500** | SfiI-subtelomeric fragment related protein family member, putative | 2.09 | $3.92 \times 10^{-15}$ | 2.16 | 0.0235 |
| **TA13345** | hypothetical protein | 2.07 | $5.14 \times 10^{-15}$ | 2.32 | 0.0250 |
| **TA19575** | hypothetical protein, conserved | 2.06 | $5.66 \times 10^{-15}$ | 2.37 | 0.0252 |
| **TA09600** | Tpr-related protein family member, putative | 2.01 | $7.70 \times 10^{-15}$ | 2.61 | 0.0275 |
| **TA03480** | hypothetical protein, conserved | 2.03 | $8.04 \times 10^{-15}$ | 2.61 | 0.0272 |
| **TA19075** | hypothetical protein | 2.03 | $8.17 \times 10^{-15}$ | 2.63 | 0.0271 |
| **TA21050** | hypothetical protein | 2.02 | $8.20 \times 10^{-15}$ | 2.63 | 0.0268 |
| **TA03855** | Tpr-related protein family member, putative | 2.03 | $9.80 \times 10^{-15}$ | 2.74 | 0.0277 |
| **TA12015** | hypothetical protein | 2.00 | $1.02 \times 10^{-14}$ | 2.79 | 0.0279 |
