# Supplementary material for: ApiAP2 Factors as Candidate Regulators of Stochastic Commitment to Merozoite Production in Theileria annulata
Source: PLoS Negl Trop Dis. 2015 Aug 14;9(8):e0003933. doi: 10.1371/journal.pntd.0003933 (PMC4537280; doi:10.1371/journal.pntd.0003933)
Supplement: S3 Table — (PDF) [file pntd.0003933.s003.pdf]

## S3 Table: Top 100 genes displaying reduced expression, macroschizont (Day 0) to merozoite stage (Day 9)

| Gene ID | Annotation | FC | RP score | EE | FDR |
|---|---|---|---|---|---|
| **TA19865** | surface protein d precursor | -3.38 | $7.04 \times 10^{-31}$ | 0.00 | 0.0000 |
| **TA11410** | Theileria-specific sub-telomeric protein, SVSP family, putative | -3.20 | $1.01 \times 10^{-28}$ | 0.00 | 0.0000 |
| **TA11405** | subtelomeric sfi-fragment-related protein family member, putative | -3.12 | $6.57 \times 10^{-28}$ | 0.00 | 0.0000 |
| **TA15705** | hypothetical protein (ta9) | -3.10 | $2.17 \times 10^{-27}$ | 0.00 | 0.0000 |
| **TA09805** | Theileria-specific sub-telomeric protein, SVSP family | -2.93 | $7.76 \times 10^{-26}$ | 0.00 | 0.0000 |
| **TA10505** | hypothetical protein | -2.91 | $1.76 \times 10^{-25}$ | 0.00 | 0.0000 |
| **TA09790** | Theileria-specific sub-telomeric protein, SVSP family | -2.69 | $8.78 \times 10^{-24}$ | 0.00 | 0.0000 |
| **TA18010** | integral membrane protein, putative | -2.69 | $1.44 \times 10^{-23}$ | 0.00 | 0.0000 |
| **TA09420** | Theileria-specific sub-telomeric protein, SVSP family, putative | -2.61 | $2.04 \times 10^{-23}$ | 0.00 | 0.0000 |
| **TA02480** | hexose transporter (HT1 homologue), putative | -2.61 | $3.93 \times 10^{-23}$ | 0.00 | 0.0000 |
| **TA05580** | Theileria-specific sub-telomeric protein, SVSP family | -2.59 | $4.48 \times 10^{-23}$ | 0.00 | 0.0000 |
| **TA15695** | hypothetical protein | -2.64 | $4.76 \times 10^{-23}$ | 0.00 | 0.0000 |
| **TA09810** | Theileria-specific sub-telomeric protein, SVSP family | -2.54 | $6.70 \times 10^{-23}$ | 0.00 | 0.0000 |
| **TA09435** | Theileria-specific sub-telomeric protein, SVSP family, putative | -2.52 | $3.11 \times 10^{-22}$ | 0.00 | 0.0000 |
| **TA15710** | hypothetical protein | -2.50 | $4.99 \times 10^{-22}$ | 0.00 | 0.0000 |
| **TA18895** | conserved Theileria-specific sub-telomeric protein, SVSP family | -2.43 | $7.76 \times 10^{-22}$ | 0.00 | 0.0000 |
| **TA09430** | Theileria-specific sub-telomeric protein, SVSP family, putative | -2.40 | $1.95 \times 10^{-21}$ | 0.01 | 0.0006 |
| **TA17555** | Theileria-specific sub-telomeric protein, SVSP family | -2.40 | $2.15 \times 10^{-21}$ | 0.01 | 0.0006 |

| | | | | | |
|---|---|---|---|---|---|
| **TA11940** | hypothetical protein | -2.39 | $2.68 \times 10^{-21}$ | 0.01 | 0.0005 |
| **TA09815** | SfiI-subtelomeric fragment related protein family member, putative | -2.36 | $3.82 \times 10^{-21}$ | 0.02 | 0.0010 |
| **TA09800** | Theileria-specific sub-telomeric protein, SVSP family | -2.37 | $4.30 \times 10^{-21}$ | 0.02 | 0.0010 |
| **TA10530** | hypothetical protein | -2.35 | $5.18 \times 10^{-21}$ | 0.02 | 0.0009 |
| **TA17545** | Theileria-specific sub-telomeric protein, SVSP family | -2.25 | $4.52 \times 10^{-20}$ | 0.05 | 0.0022 |
| **TA05575** | Theileria-specific sub-telomeric protein, SVSP family | -2.24 | $5.32 \times 10^{-20}$ | 0.05 | 0.0021 |
| **TA19005** | conserved Theileria-specific sub-telomeric protein, SVSP family | -2.23 | $7.05 \times 10^{-20}$ | 0.07 | 0.0028 |
| **TA17125** | Theileria-specific sub-telomeric protein, SVSP family | -2.19 | $1.21 \times 10^{-19}$ | 0.08 | 0.0031 |
| **TA09505** | SfiI-subtelomeric fragment related protein family member, putative | -2.18 | $2.01 \times 10^{-19}$ | 0.09 | 0.0033 |
| **TA18890** | conserved Theileria-specific sub-telomeric protein, SVSP family | -2.17 | $2.72 \times 10^{-19}$ | 0.09 | 0.0032 |
| **TA20095** | Tashat2 protein | -2.13 | $6.55 \times 10^{-19}$ | 0.15 | 0.0052 |
| **TA02905** | hypothetical protein | -2.04 | $8.61 \times 10^{-19}$ | 0.16 | 0.0053 |
| **TA16035** | Theileria-specific sub-telomeric protein, SVSP family, putative | -2.09 | $8.76 \times 10^{-19}$ | 0.16 | 0.0052 |
| **TA16030** | Theileria-specific sub-telomeric protein, SVSP family, putative | -2.07 | $1.10 \times 10^{-18}$ | 0.17 | 0.0053 |
| **TA09510** | SfiI-subtelomeric fragment related protein family member, putative | -1.98 | $1.21 \times 10^{-18}$ | 0.17 | 0.0052 |
| **TA12265** | SfiI-subtelomeric fragment related protein family member, putative | -2.08 | $1.23 \times 10^{-18}$ | 0.17 | 0.0050 |
| **TA11945** | hypothetical protein | -2.06 | $1.73 \times 10^{-18}$ | 0.19 | 0.0054 |
| **TA04675** | hypothetical protein | -1.98 | $1.93 \times 10^{-18}$ | 0.19 | 0.0053 |
| **TA11400** | SfiI-subtelomeric fragment related protein family member, putative | -2.05 | $2.20 \times 10^{-18}$ | 0.20 | 0.0054 |
| **TA16090** | glutenin, putative | -1.97 | $2.95 \times 10^{-18}$ | 0.21 | 0.0055 |

| | | | | | |
|---|---|---|---|---|---|
| **TA17485** | hypothetical protein | -1.90 | $4.18 \times 10^{-18}$ | 0.21 | 0.0054 |
| **TA05540** | Theileria-specific sub-telomeric protein, SVSP family | -2.01 | $5.41 \times 10^{-18}$ | 0.23 | 0.0058 |
| **TA10420** | hypothetical protein | -1.93 | $5.90 \times 10^{-18}$ | 0.23 | 0.0056 |
| **TA05960** | Tpr-related protein family member, putative | -1.88 | $7.87 \times 10^{-18}$ | 0.28 | 0.0067 |
| **TA18950** | conserved Theileria-specific sub-telomeric protein, SVSP family | -1.96 | $1.15 \times 10^{-17}$ | 0.30 | 0.0070 |
| **TA15015** | Tpr-related protein family member, putative | -1.94 | $1.31 \times 10^{-17}$ | 0.30 | 0.0068 |
| **TA05565** | Theileria-specific sub-telomeric protein, SVSP family | -1.93 | $2.12 \times 10^{-17}$ | 0.37 | 0.0082 |
| **TA18865** | conserved Theileria-specific sub-telomeric protein, SVSP family | -1.93 | $2.15 \times 10^{-17}$ | 0.37 | 0.0080 |
| **TA17915** | hypothetical protein | -1.82 | $2.96 \times 10^{-17}$ | 0.43 | 0.0091 |
| **TA16025** | Theileria-specific sub-telomeric protein, SVSP family, putative | -1.91 | $3.02 \times 10^{-17}$ | 0.43 | 0.0090 |
| **TA07475** | SfiI-subtelomeric fragment related protein family member, putative | -1.81 | $3.60 \times 10^{-17}$ | 0.49 | 0.0100 |
| **TA18540** | hypothetical protein | -1.77 | $3.99 \times 10^{-17}$ | 0.51 | 0.0102 |
| **TA05150** | hypothetical protein, conserved | -1.87 | $5.19 \times 10^{-17}$ | 0.54 | 0.0106 |
| **TA17375** | polymorphic antigen precursor, putative | -1.79 | $5.83 \times 10^{-17}$ | 0.56 | 0.0108 |
| **TA11950** | hypothetical P-, Q-rich protein family protein, putative | -1.78 | $6.87 \times 10^{-17}$ | 0.56 | 0.0106 |
| **TA16185** | haloacid dehalogenase-like family hydrolase, putative | -1.78 | $7.17 \times 10^{-17}$ | 0.59 | 0.0109 |
| **TA09425** | Theileria-specific sub-telomeric protein, SVSP family, putative | -1.84 | $9.61 \times 10^{-17}$ | 0.67 | 0.0122 |
| **TA11385** | Theileria-specific sub-telomeric protein, SVSP family, putative | -1.84 | $1.03 \times 10^{-16}$ | 0.67 | 0.0120 |
| **TA17120** | Theileria-specific sub-telomeric protein, SVSP family | -1.83 | $1.34 \times 10^{-16}$ | 0.71 | 0.0125 |
| **TA04470** | hypothetical protein | -1.72 | $1.68 \times 10^{-16}$ | 0.81 | 0.0140 |
| **TA05560** | Theileria-specific sub-telomeric protein, SVSP family | -1.77 | $2.21 \times 10^{-16}$ | 0.84 | 0.0142 |

| | | | | | |
|---|---|---|---|---|---|
| **TA19060** | conserved Theileria-specific sub-telomeric protein, SVSP family | -1.72 | $2.22 \times 10^{-16}$ | 0.84 | 0.0140 |
| **TA05965** | Tpr-related protein family member, putative | -1.65 | $2.22 \times 10^{-16}$ | 0.84 | 0.0138 |
| **TA05555** | Theileria-specific sub-telomeric protein, SVSP family | -1.77 | $2.31 \times 10^{-16}$ | 0.84 | 0.0135 |
| **TA10655** | hypothetical protein, conserved | -1.71 | $2.50 \times 10^{-16}$ | 0.85 | 0.0135 |
| **TA17550** | Theileria-specific sub-telomeric protein, SVSP family | -1.78 | $2.56 \times 10^{-16}$ | 0.87 | 0.0136 |
| **TA05570** | Theileria-specific sub-telomeric protein, SVSP family | -1.79 | $2.64 \times 10^{-16}$ | 0.89 | 0.0137 |
| **TA03125** | Tash1-like protein, putative | -1.69 | $2.89 \times 10^{-16}$ | 0.94 | 0.0142 |
| **TA11415** | SfiI-subtelomeric fragment related protein family member, putative | -1.77 | $3.73 \times 10^{-16}$ | 1.01 | 0.0151 |
| **TA17540** | Theileria-specific sub-telomeric protein, SVSP family | -1.76 | $3.77 \times 10^{-16}$ | 1.01 | 0.0149 |
| **TA13350** | hypothetical protein | -1.74 | $4.01 \times 10^{-16}$ | 1.01 | 0.0146 |
| **TA04895** | hypothetical protein, conserved | -1.61 | $4.98 \times 10^{-16}$ | 1.05 | 0.0150 |
| **TA15580** | hypothetical protein | -1.57 | $5.57 \times 10^{-16}$ | 1.14 | 0.0161 |
| **TA11935** | hypothetical protein | -1.75 | $5.69 \times 10^{-16}$ | 1.16 | 0.0161 |
| **TA03120** | Tash-like protein, putative | -1.64 | $5.84 \times 10^{-16}$ | 1.17 | 0.0160 |
| **TA16555** | hypothetical protein | -1.62 | $6.38 \times 10^{-16}$ | 1.19 | 0.0161 |
| **TA05545** | Theileria-specific sub-telomeric protein, SVSP family | -1.72 | $8.30 \times 10^{-16}$ | 1.29 | 0.0172 |
| **TA13965** | hypothetical protein | -1.55 | $1.14 \times 10^{-15}$ | 1.46 | 0.0192 |
| **TA09785** | Theileria-specific sub-telomeric protein, SVSP family | -1.70 | $1.30 \times 10^{-15}$ | 1.54 | 0.0200 |
| **TA17535** | Theileria-specific sub-telomeric protein, SVSP family | -1.64 | $1.31 \times 10^{-15}$ | 1.55 | 0.0199 |
| **TA16820** | hypothetical protein | -1.57 | $1.54 \times 10^{-15}$ | 1.62 | 0.0205 |
| **TA10865** | Tpr-related protein family member, putative | -1.55 | $1.56 \times 10^{-15}$ | 1.64 | 0.0205 |
| **TA03535** | hypothetical protein | -1.63 | $1.61 \times 10^{-15}$ | 1.65 | 0.0204 |
| **TA09865** | Theileria-specific sub-telomeric | -1.60 | $2.04 \times 10^{-15}$ | 1.77 | 0.0216 |

| | | | | | |
|---|---|---|---|---|---|
| | protein, SVSP family | | | | |
| **TA12370** | hypothetical protein, conserved | -1.52 | $2.49 \times 10^{-15}$ | 1.92 | 0.0231 |
| **TA17346** | hypothetical protein | -1.48 | $3.44 \times 10^{-15}$ | 2.12 | 0.0252 |
| **TA04550** | hypothetical protein | -1.49 | $3.81 \times 10^{-15}$ | 2.14 | 0.0252 |
| **TA10735** | GATA-specific transcription factor, putative | -1.50 | $4.85 \times 10^{-15}$ | 2.28 | 0.0265 |
| **TA10755** | hypothetical protein, conserved | -1.47 | $5.10 \times 10^{-15}$ | 2.32 | 0.0267 |
| **TA09025** | hypothetical protein, conserved | -1.45 | $5.73 \times 10^{-15}$ | 2.37 | 0.0269 |
| **TA03145** | Tash1-like protein, putative | -1.53 | $5.91 \times 10^{-15}$ | 2.39 | 0.0269 |
| **TA08365** | hypothetical protein | -1.44 | $7.29 \times 10^{-15}$ | 2.54 | 0.0282 |
| **TA12635** | Theileria-specific hypothetical protein family member, putative | -1.43 | $7.89 \times 10^{-15}$ | 2.61 | 0.0287 |
| **TA09795** | Theileria-specific sub-telomeric protein, SVSP family | -1.56 | $9.30 \times 10^{-15}$ | 2.72 | 0.0296 |
| **TA11390** | Theileria-specific sub-telomeric protein, SVSP family, putative | -1.47 | $1.43 \times 10^{-14}$ | 3.07 | 0.0330 |
| **TA11960** | hypothetical P-,Q-rich family protein, putative | -1.41 | $1.88 \times 10^{-14}$ | 3.34 | 0.0355 |
| **TA10770** | hypothetical protein, conserved | -1.36 | $2.18 \times 10^{-14}$ | 3.51 | 0.0369 |
| **TA12945** | hypothetical protein, conserved | -1.39 | $2.32 \times 10^{-14}$ | 3.54 | 0.0369 |
| **TA12525** | Theileria-specific hypothetical protein family member, putative | -1.35 | $2.87 \times 10^{-14}$ | 3.75 | 0.0387 |
| **TA06330** | Tpr-related protein family member, putative | -1.35 | $3.54 \times 10^{-14}$ | 4.08 | 0.0416 |
| **TA06060** | hypothetical protein, conserved | -1.35 | $3.79 \times 10^{-14}$ | 4.26 | 0.0430 |
| **TA10800** | hypothetical protein, conserved | -1.35 | $4.20 \times 10^{-14}$ | 4.39 | 0.0439 |
