# Supplementary material for: ApiAP2 Factors as Candidate Regulators of Stochastic Commitment to Merozoite Production in Theileria annulata
Source: PLoS Negl Trop Dis. 2015 Aug 14;9(8):e0003933. doi: 10.1371/journal.pntd.0003933 (PMC4537280; doi:10.1371/journal.pntd.0003933)
Supplement: S4 Table — Differential expression between cell lines was identified by RP analysis of microarray data (FDR < 0.05). Genes encoding AP2 domains predicted to bind (A)CACAC(A) motifs are highlighted with a blue bar. (PDF) [file pntd.0003933.s004.pdf]

S4 Table: Differential expression of TaApiAP2 genes between infected cell lines, D7 versus D7B12

Gene ID	D7 vs D7B12 Fold change	D7 vs D7B12 False discovery rate
TA07550	3.23	0.01
TA13395	1.10	1.20
TA07100	1.13	1.21
TA19920	1.13	1.21
TA18095	1.14	1.21
TA02615	1.51	0.82
TA09965	1.55	0.75
TA16535	1.66	0.49
TA11665	2.00	0.21
TA11145	3.95	0.01
TA04435	7.38	0.00
TA16485	1.45	0.81
TA17415	1.56	0.74
TA16105	2.42	0.06
TA08375	1.72	0.47
TA12015	1.79	0.41
TA05055	1.51	0.79
TA04145	1.00	1.02
TA13515	1.87	0.32
