# Supplementary figures and images for: ApiAP2 Factors as Candidate Regulators of Stochastic Commitment to Merozoite Production in Theileria annulata
Source: PLoS Negl Trop Dis. 2015 Aug 14;9(8):e0003933. doi: 10.1371/journal.pntd.0003933 (PMC4537280; doi:10.1371/journal.pntd.0003933)

S1 Figure: qRT-PCR expression profile of *TA15705*

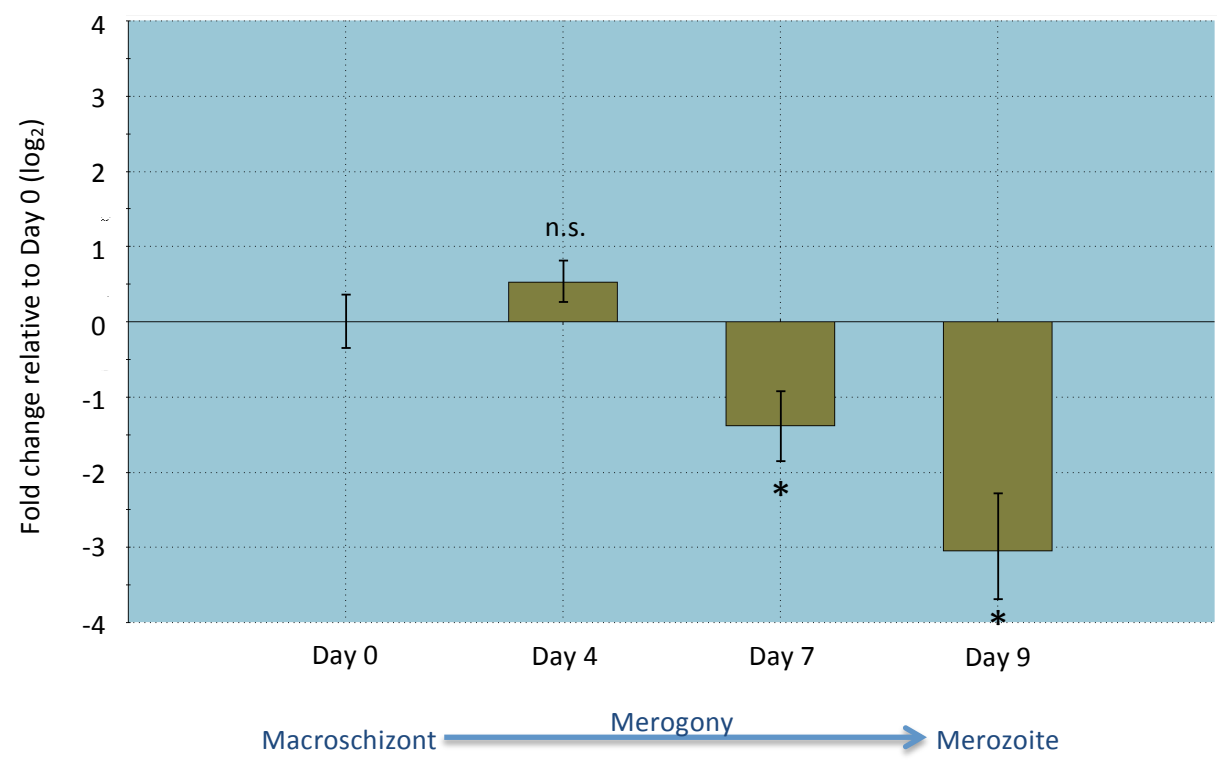

Supplement: S1 Fig — (PDF) [file pntd.0003933.s005.pdf]

## S4 Figure: EMSA of GST-AP2 domain fusion protein TA16485

Probe: TCTATA core motif

1 2 3 4

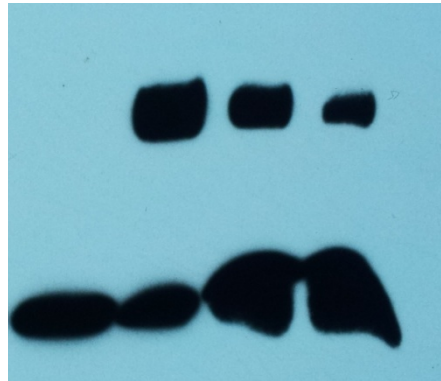

Supplement: S4 Fig — (PDF) [file pntd.0003933.s008.pdf]

**S5 Figure: EMSA of GST-AP2 domain fusion protein TA12015**

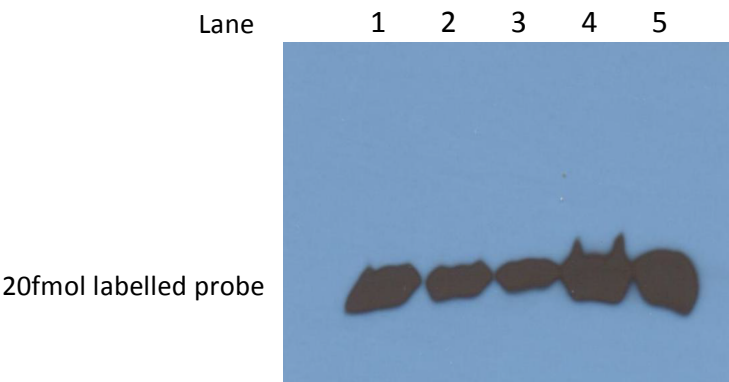

Supplement: S5 Fig — (PDF) [file pntd.0003933.s009.pdf]

**S6 Figure: EMSA of double (A)CACAC(A) motif probe and nuclear extract from uninfected BL20 and PNE, Day 9**

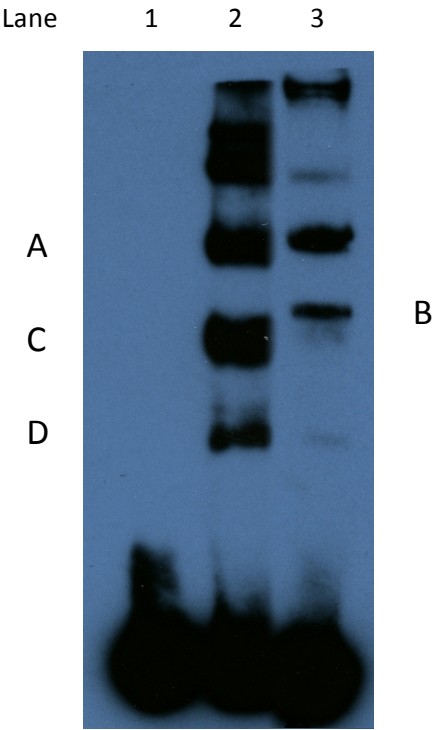

Supplement: S6 Fig — Letters denote shift positions detected in both infected and uninfected cells at Day 0 (37°C), shift B was only detected in extracts derived from infected cells undergoing merogony (Day 9, 41°C). (PDF) [file pntd.0003933.s010.pdf]

Figure S9: qRTR PCR data for ApiAP2 domainR encoding gene *TA13515* (TaAP2.g)

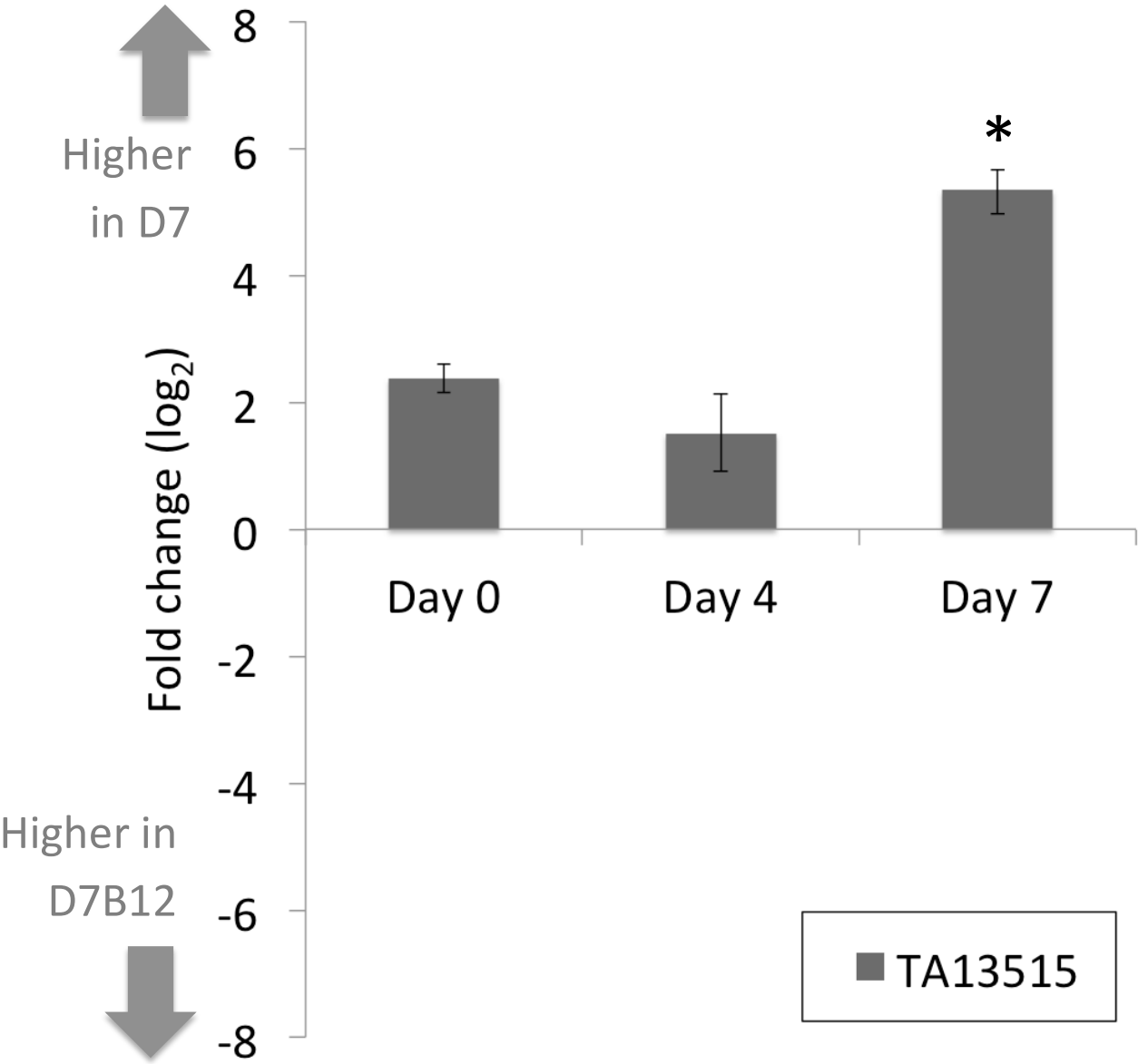

Supplement: S9 Fig — Fold-change in expression between cell lines was computed at Day 0 (macroschizont) and Day 4 and Day 7 points of a time-course of differentiation to the merozoite; * denotes significant (P value ≤ 0.05) fold-change elevated expression in D7 vs D7B12. (PDF) [file pntd.0003933.s013.pdf]

**S10 Figure: EMSA of GSTR AP2 domain fusion proteins:  
TA11145, TA13515, TA12015 and TA16485**

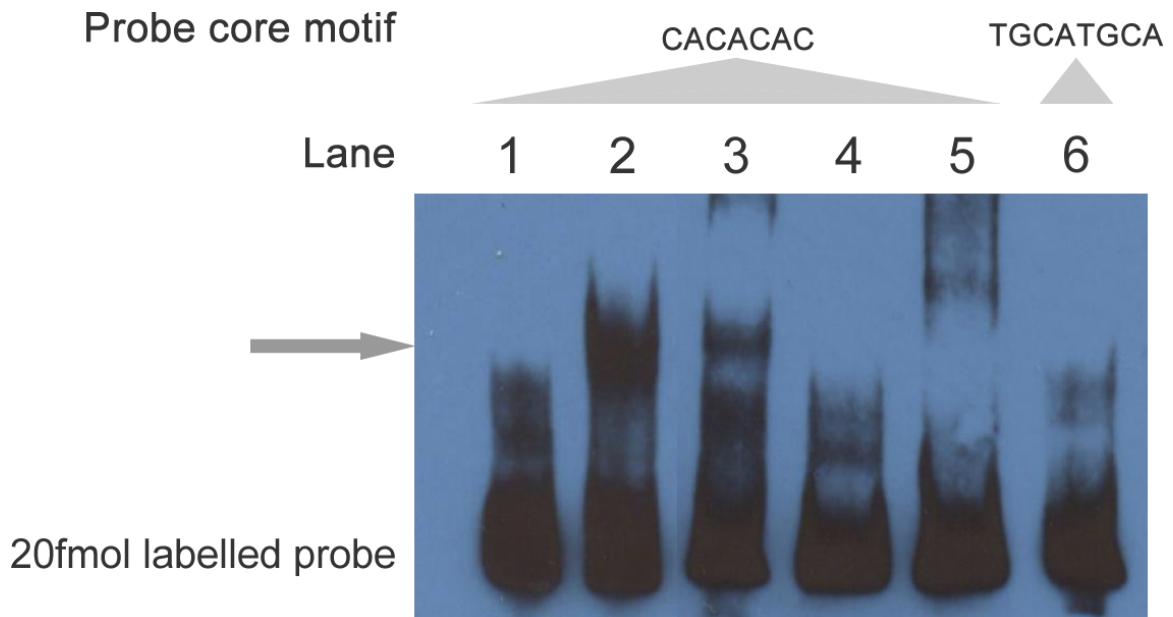

Supplement: S10 Fig — Arrow denotes the shift position obtained with GST-TA11145D, the more minor shift obtained with GST-TA13515D may be indicative weaker/partial recognition of the probe. (PDF) [file pntd.0003933.s014.pdf]
