# Supplementary material for: ApiAP2 Factors as Candidate Regulators of Stochastic Commitment to Merozoite Production in Theileria annulata
Source: PLoS Negl Trop Dis. 2015 Aug 14;9(8):e0003933. doi: 10.1371/journal.pntd.0003933 (PMC4537280; doi:10.1371/journal.pntd.0003933)
Supplement: S2 Fig — Regions of predicted secondary structure are indicated above the alignment and were predicted with Phyre2 using three independent secondary structure prediction programs: Psi-Pred [58], SSPro [59] and JNet [60]. * identity,:. similarity. (PDF) [file pntd.0003933.s006.pdf]

S2 Figure: Alignment of paralogous AP2 domains encoded by four genes upregulated during merogony in *T. annulata*

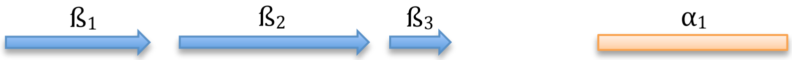


```
TA16485     PKIRGVWFNSTVRRMGW---VGQAYKKCKRIEKIFSINKHGFEGARKLAIAFRNS-
TA12015     SDVSGVYYH--FKKMEWRTICKDPFNNSKRTQKTFGINKYGFYEAKHRAEMTAFEI
TA11145     -KVSGVWYD--TNRHLWR---VVYMKGNKRKTQGFSSLKLGYEEARRKAIQMRHEM
TA13515     --IHCVWRDVNRGHCSWR---CRWWENGKRLSKNFNVKRFGEFEAMRMAITMKIR-
              :  *: .    :  *        :  **  : *.  : *   * : *
```
