# Supplementary material for: ApiAP2 Factors as Candidate Regulators of Stochastic Commitment to Merozoite Production in Theileria annulata
Source: PLoS Negl Trop Dis. 2015 Aug 14;9(8):e0003933. doi: 10.1371/journal.pntd.0003933 (PMC4537280; doi:10.1371/journal.pntd.0003933)

# S3 Figure: Correlation plots of ApiAP2 gene expression and putative target genes of the binding domain

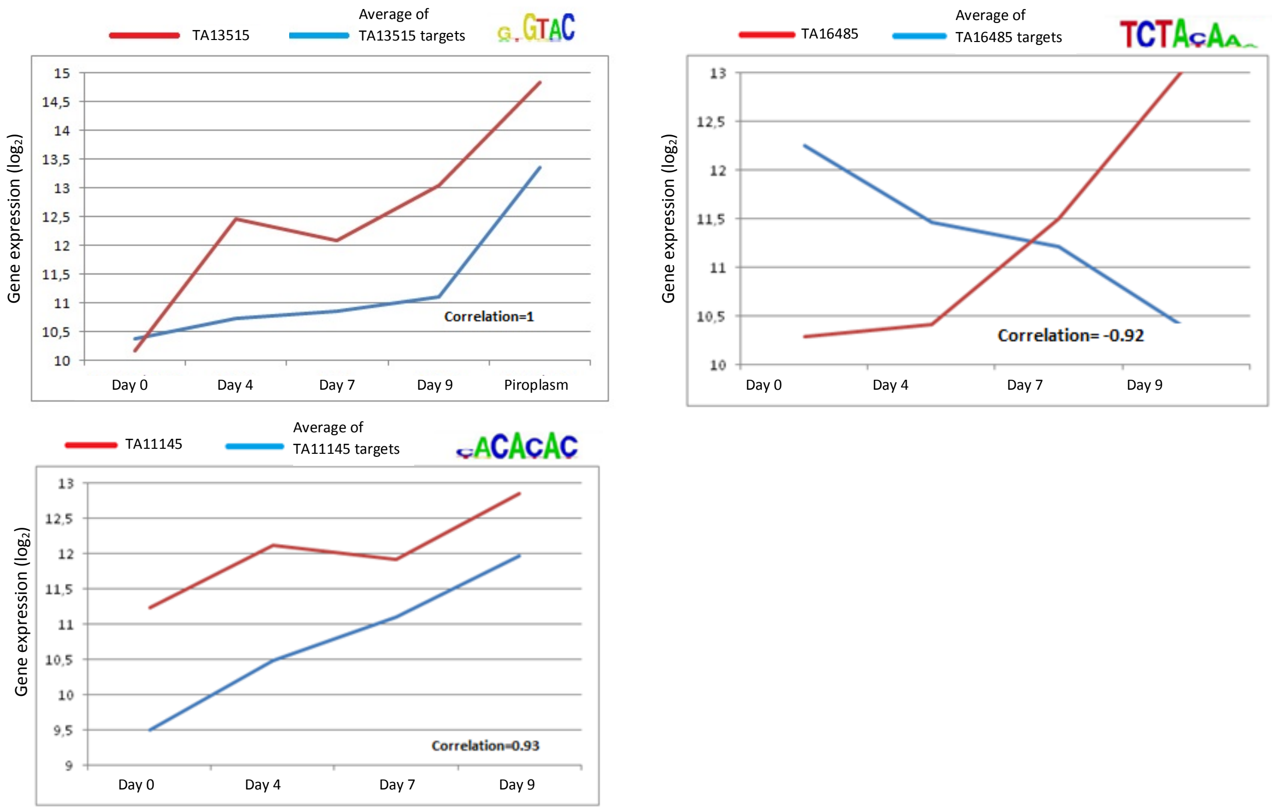

Supplement: S3 Fig — ApiAP2 gene expression profile is shown in red, while the average profile of putative target genes possessing the motif bound by the P. falciparum orthologous domain are shown in blue, a significant Pearson correlation coefficient value is indicated for each plot. (PDF) [file pntd.0003933.s007.pdf]
