# Supplementary material for: ApiAP2 Factors as Candidate Regulators of Stochastic Commitment to Merozoite Production in Theileria annulata
Source: PLoS Negl Trop Dis. 2015 Aug 14;9(8):e0003933. doi: 10.1371/journal.pntd.0003933 (PMC4537280; doi:10.1371/journal.pntd.0003933)
Supplement: S7 Fig — (PDF) [file pntd.0003933.s011.pdf]

S7 Figure: Region upstream of AP2 domain gene *TA11145*

TTTTCCCAAATTTCTTAATTTATTTTATTCAAAATTTAGTTAAACATATATCTTATTTTA
AGAAAAATATAGAACATAAATGTGTAAATTAATCTTGAAGTTTTTGATAATATTATGGAA
GAAGAT**ACACAC**TTATG**CACACA**CAGATTCTTTTCAAAAACACCAAGGTATATGAATGGA
TTATGTGTCTTAACAAGGAATAATTAACAAAAATGATTATTTAAGGTTAAATTATTGGAA
AATTAATAAGTTTATAAGAAATAAATAGAATGTGTAAGATGAAT**ACACAC**TTTGAAATTT
TGATTTAAAAATTGAAAATTAACAGAAAATATTTTAAAAAATGTAAAATAACACAATAAA
AAGTGGTCAAAACGATAGAATTAAGGAAATTCTTGAGAAATGATTGATTTAGATTTAATG
TGTAGAAAAAGAAAGGGAAATTCTCAAGGAAACAAGGACCCAAAAGTCTGGAAGACAGGA
CATTTTAATAACATTATAAATTATATTAAGATAGAAACATAATAATTTTCTAATGATGGA
AGGAATTCAAGGACTTTACAGAATAACATAATATTGGGAGATTCGGAGAATGGAATTTGG
GGCGTGAGCCATATTAACTTGTCTGCATTCACGAATATTTAAATAACTGCTTAACACTGA
TTCTCTCAAGTGTTAACATTAGTTTATACACTATATTTTCTATAATTAATATTTAATTTT
CACCTCTAGTATCCATTTTATTAGGTGTCGTGAAACACCTCTGTTCCTTCACTTGACACT
TTTAATTTCTCGTACACATCGTTAAACTTATACAATAAAAAGGATATAAACAATTTGATA
ATTAATTTAAACCACAAAGATACGGTATTTGTAAGCCCTTTTATTTC**TGTGTG**ACCACGA
TGGCGACTTGCCTTTTTTAGATTTTCCCAAAACATTTCTTCATTATTTTAATTTAAAATA
TTTTGGCAAAGTATCACGTGTAATTCCCAAAATATTTATAGTATTAATTTTTGGTATTTC
TCTTTAATGCAATCACTGTAATATTTTGTATTTTAATAATTTAAACCTGGAGCCGACATT
GTTCAAAATTGGATCATAACATTCGCTTTGAATGATAGTTGCTAATATTTTAATAAGATG
ACCTTTTAAAACTCATATAACCCCCGAGATTTGGTTACAGATA**ACACAC**TTTAAATCTTG
TAACAAAGTTAGCTGGAAGGGTTAATGTCGAGTACGTACGCCTCACTTCTTAAAACAATA
CAATAATTAATGAACTTTAATAATAACACAAAACAAGTGTCAATTGATGACAAATGATAC
ACATAAGA**CACACA**GTTATGAGTATCA**ACACACA**AATGTATTAATAGACAAACTTACTGA
GAAAATAACTATATAGAATAAAACTAACCTACAATTCACTGAGAAATTTTAATTTAAATA
TAAAACTGAAGTTTAATTTTATCAACATAGAAATGAAATTTGAGGTTTTGTAATTTTAAA
TCTCAAGTTTATATATTTATCATG
