# Supplementary material for: ApiAP2 Factors as Candidate Regulators of Stochastic Commitment to Merozoite Production in Theileria annulata
Source: PLoS Negl Trop Dis. 2015 Aug 14;9(8):e0003933. doi: 10.1371/journal.pntd.0003933 (PMC4537280; doi:10.1371/journal.pntd.0003933)

S8 Figure: Alignment of ApiAP2 domain encoded by *TA07100* with orthologues in other *Theileria*, *Plasmodium* and *Babesia*

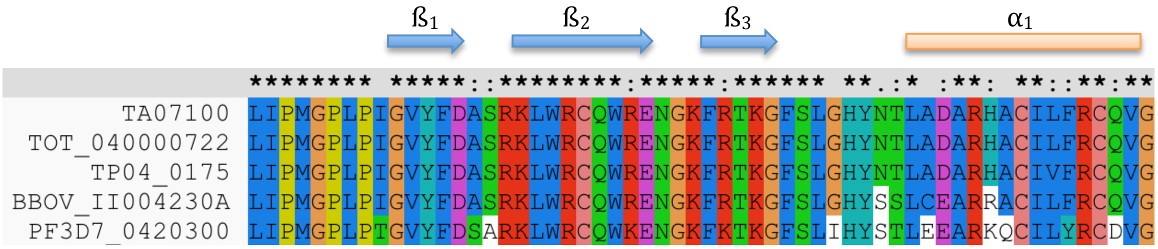

Supplement: S8 Fig — The domain in P. falciparum has been shown to bind an (A)CACAC(A) type motif. Regions of predicted secondary structure are indicated above the alignment and were predicted with Phyre2 using three independent secondary structure prediction programs: Psi-Pred [58], SSPro [59] and JNet [60]. * identity,:. similarity. (PDF) [file pntd.0003933.s012.pdf]
